# SARS-CoV-2 outbreak in a tri-national urban area is dominated by a B.1 lineage variant linked to a mass gathering event

**DOI:** 10.1371/journal.ppat.1009374

**Published:** 2021-03-19

**Authors:** Madlen Stange, Alfredo Mari, Tim Roloff, Helena MB Seth-Smith, Michael Schweitzer, Myrta Brunner, Karoline Leuzinger, Kirstine K. Søgaard, Alexander Gensch, Sarah Tschudin-Sutter, Simon Fuchs, Julia Bielicki, Hans Pargger, Martin Siegemund, Christian H. Nickel, Roland Bingisser, Michael Osthoff, Stefano Bassetti, Rita Schneider-Sliwa, Manuel Battegay, Hans H. Hirsch, Adrian Egli

**Affiliations:** 1 Applied Microbiology Research, Department of Biomedicine, University of Basel, Basel, Switzerland; 2 Clinical Bacteriology and Mycology, University Hospital Basel, Basel, Switzerland; 3 Swiss Institute for Bioinformatics, Basel, Switzerland; 4 Human Geography, University of Basel, Basel, Switzerland; 5 Clinical Virology, University Hospital Basel, Basel, Switzerland; 6 Transplantation & Clinical Virology, Department of Biomedicine, University of Basel, Basel, Switzerland; 7 Infectious Diseases and Hospital Epidemiology, University Hospital Basel and University of Basel, Basel, Switzerland; 8 Health Services for the City of Basel, Basel, Switzerland; 9 Pediatric Infectious Diseases, University Children’s Hospital Basel, Basel, Switzerland; 10 Intensive Care Unit, University Hospital Basel, Basel, Switzerland; 11 Emergency Medicine, University Hospital Basel, Basel, Switzerland; 12 Internal Medicine, University Hospital Basel, Basel, Switzerland; The Peter Doherty Institute and Melbourne University, AUSTRALIA

## Abstract

The first case of SARS-CoV-2 in Basel, Switzerland was detected on February 26^th^ 2020. We present a phylogenetic study to explore viral introduction and evolution during the exponential early phase of the local COVID-19 outbreak from February 26^th^ until March 23^rd^. We sequenced SARS-CoV-2 naso-oropharyngeal swabs from 746 positive tests that were performed at the University Hospital Basel during the study period. We successfully generated 468 high quality genomes from unique patients and called variants with our COVID-19 Pipeline (COVGAP), and analysed viral genetic diversity using PANGOLIN taxonomic lineages. To identify introduction and dissemination events we incorporated global SARS-CoV-2 genomes and inferred a time-calibrated phylogeny. Epidemiological data from patient questionnaires was used to facilitate the interpretation of phylogenetic observations. The early outbreak in Basel was dominated by lineage B.1 (83·6%), detected first on March 2^nd^, although the first sample identified belonged to B.1.1. Within B.1, 68·2% of our samples fall within a clade defined by the SNP C15324T (‘Basel cluster’), including 157 identical sequences at the root of the ‘Basel cluster’, some of which we can specifically trace to regional spreading events. We infer the origin of B.1-C15324T to mid-February in our tri-national region. The other genomes map broadly over the global phylogenetic tree, showing several introduction events from and/or dissemination to other regions of the world via travellers. Family transmissions can also be traced in our data. A single lineage variant dominated the outbreak in the Basel area while other lineages, such as the first (B.1.1), did not propagate. A mass gathering event was the predominant initial source of cases, with travel returners and family transmissions to a lesser extent. We highlight the importance of adding specific questions to epidemiological questionnaires, to obtain data on attendance of large gatherings and their locations, as well as travel history, to effectively identify routes of transmissions in up-coming outbreaks. This phylogenetic analysis in concert with epidemiological and contact tracing data, allows connection and interpretation of events, and can inform public health interventions.

**Trial Registration:** ClinicalTrials.gov NCT04351503.

## Author summary

COVID-19 was first reported in December 2019 in Wuhan, China, and has spread around the globe since. The most common tool for tracking and containing the spread of the disease-causing virus–SARS-CoV-2 –are classical epidemiology and contact tracing, which collapse under too high case burdens as seen in this pandemic. However, whole-genome sequencing and phylogenetic analyses of the viral genomes will be crucial to understand the virus evolution and outbreak dynamics to ultimately stop the spread of the disease. Here we combined epidemiological data and whole-genome sequences originating from the time of the arrival of the virus in early 2020 in a typical medium-sized European city, namely Basel-City, Switzerland. On one hand, we identified limited community spread of very distinct viral variants originating from travel returners, which is possibly due to the heightened attention and restrictions imposed on international travellers. On the other hand, we find evidence for large cryptic community spread of a single virus variant in the city, which we infer to have originated around mid-February 2020 in the larger tri-national area around Basel, and which has epidemiological links to a mass gathering event in the region of Alsace, France.

## Introduction

The COVID-19 pandemic rapidly spread around the globe during the first six months of 2020. The causative coronavirus, SARS-CoV-2, is the subject of many studies using genomic analysis providing key insights into viral diversity across cities [1], provinces [2–5], countries [6–11], and globally [12]. SARS-CoV-2 has an estimated substitution rate of 0.71–1.40x10^-3^ [13], which translates to 21–42 single nucleotide polymorphisms (SNPs) per year. Due to the accumulation of mutations, phylogenetic analysis of SARS-CoV-2 is becoming more granular over time [14], providing increasing resolution of transmission dynamics and events. Comparisons of SNPs within sequenced genomes allows us to explore transmission events with highest resolution across communities. The identification of transmission routes is important, especially as various public health measures are introduced, such as lockdown policies, which have been implemented on country or regional levels to limit viral transmission. The impact of public health measures can be monitored through phylogenetic analysis [3]. Genomic data can also deliver insights into the effect of specific mutations on virulence or adaptation to novel hosts. The spike protein D614G mutation, for example, has been implicated in more effective transmission [15], although the actual impact may be through fixation in an expanding lineage rather than conferring increased transmissibility per se [16].

The scope of this study is to provide a more granular picture of the phylogenetic diversification and propagation of the early-stage SARS-CoV-2 pandemic on a local scale. The City of Basel has a population of 175,350 inhabitants (median over the past five years), and half a million people live in the wider Basel area. Situated in North-Western Switzerland, directly bordering both Germany and France, Basel has almost 34,000 workers commuting daily across the international borders [17]. Given this large exchange of people in this tri-national region, the fact that the neighbouring region Alsace, France, was already experiencing an intense epidemic [18], and a low threshold testing strategy implemented weeks before the first case, we aim to explore the early stage of SARS-CoV-2 transmission dynamics in Basel and the surrounding area from the first case to one week post border closure.

## Results

### Characteristics of the longitudinal study

This cohort study includes all patient samples from Basel-City and the surrounding area during the initial 26 days of the local outbreak, between February 26^th^ and March 23^rd^ 2020. Only single, non-repeated tests per patient were considered eligible for phylogenetic analysis. This timeframe begins with the first two positively tested cases in Basel on February 26^th^ 2020, which we were able to capture via early implementation of PCR-based detection by routine diagnostics, until the date of border closure plus seven days (March 23^rd^).

**Fig 1:**
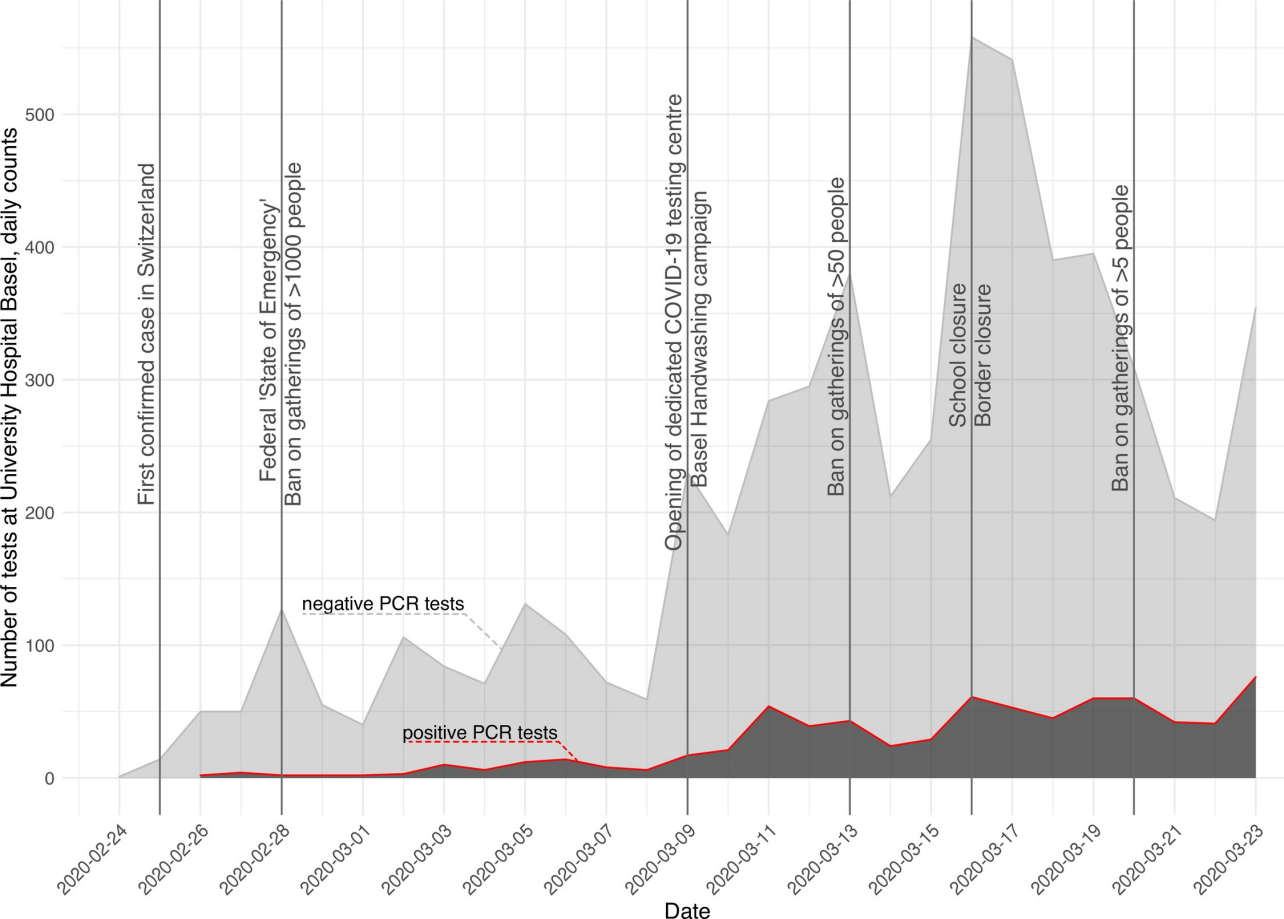
Epidemiological curve of the first COVID-19 wave in Basel-City and region, Switzerland. Positive (red line, dark grey area) and negative (grey line, light grey area) SARS-CoV-2 PCR tests are depicted, from the beginning of the outbreak in February to March 23^rd^ 2020. Major events and imposed restrictions are marked by horizontal lines. The first confirmed cases in Switzerland and Basel were on February 25^th^ and February 26^th^, respectively.

Between February 26^th^ 2020 and March 23^rd^ we performed 6,943 diagnostic PCR tests. Of these, 746 samples (10.7%) were SARS-CoV-2 positive (**Fig 1**). March 23^rd^ had the maximum number of positive tests, with 66 cases. Of all PCR tested patients and positively tested patients during the study period, a majority (55.8%) were female with a median age of 42 and 49 years, respectively (**Table 1**). Of the PCR-confirmed cases, only 17 (2.3%) were in patients younger than 18 years. (**Table 1** and S1 **Fig**). 418 (56%) patients were living in the canton of Basel-City (City of Basel, Riehen, Bettingen) and 328 (44%) were from the surrounding area.

**Table 1:** Number and age summary of all tested patients, positively tested patients, and patients with successfully sequenced SARS-CoV-2 genomes, by sex, until March 23^rd^ 2020.

		Number	%	Median Age [years]	IQR [years]	< 18 years old
All tests*	Males	3067	44.2	44	31–60	396 (5.7%)
	Females	3867	55.8	42	29–56	
Positive tested*	Males	363	48.7	49	33–61.5	17 (2.3%)
	Females	383	51.3	47	32–60	
In study cohort*	Males	222	48.1	49	34–60	12 (2.6%)
	Females	240	51.9	47	33–60	

* six patients with no information regarding sex

### COVGAP pipeline validation

We developed an in-house quality control, mapping, variant-calling and consensus generating pipeline for Illumina data, COVGAP, which we validated with *in silico* generated mock genomes to determine that no false positive variants were called (**Tables 2 and S1 and** S2 **Fig**). This validation allowed us to determine the specificity (100%), sensitivity (94.2%), and accuracy (100%) of COVGAP, thus confirming its accuracy in SNP detection, as well as its ability to call the majority of indels from short read data. Ambiguous mapping at read ends lead to insufficient coverage (<70%) and hence failure to reliably call three indels.

**Table 2:** Sensitivity, specificity, and accuracy of COVGAP.

	MOCK POSITIVE	MOCK NEGATIVE
**COVGAP POSITIVE**	TP = 180	FN = 11
**COVGAP NEGATIVE**	FP = 0	TN = 2541564

TP: true positive, FP: false positive, FN: false negative. Numbers represent cumulative counts of bases that were or were not mutated over the 16 test genomes.

Of the 746 positive SARS-CoV-2 samples, the COVGAP pipeline produced 468 (63%) high quality genomes for subsequent analysis,. The remaining samples were either not available (N = 57), did not pass sequencing quality control (N = 156), or were duplicates from the same patient (N = 65). The 468 sequenced samples are subsequently referred to as the Basel area cohort. Of these, 240 (51.9%) were from female patients and 12 (2.6%) were from patients younger than 18 years (**Table 1** and S1 Fig).

### Phylogenetic lineages observed over time in the Basel area cohort

Over the 27-day study period, 13 out of 91 globally contemporary circulating phylogenetic lineages were recorded in the Basel area; this was similar to the results across all Switzerland with only one additional lineage recorded in all Swiss sequences.

**Fig 2:**
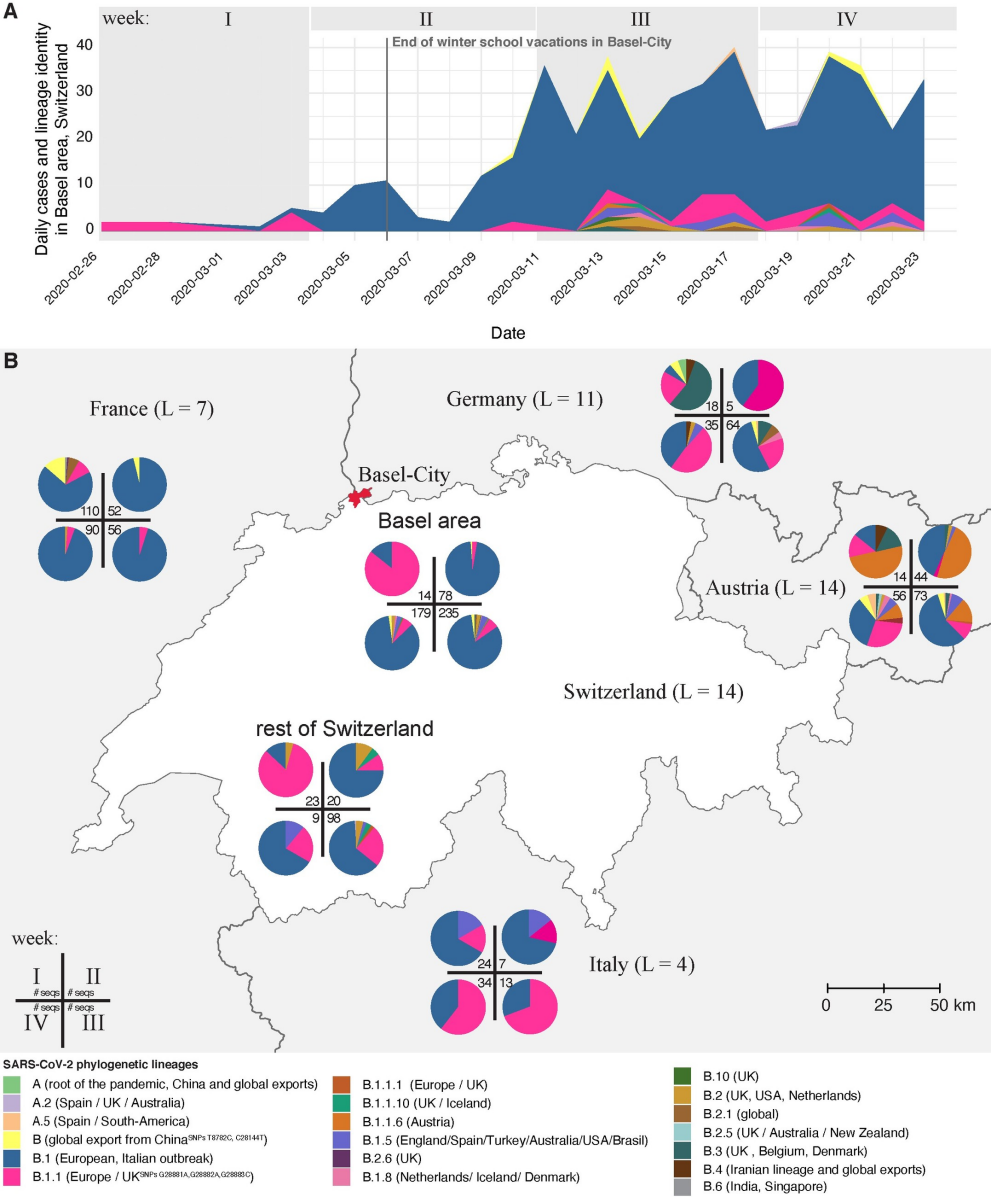
SARS-CoV-2 lineage diversity in Switzerland and neighbouring countries during the study period from the first detected case on February 26^th^ to March 23^rd^ 2020, divided into four weeks (I: February 26^th^–March 3^rd^, II: March 4^th^ - 10^th^, III: March 11^th^ - 17^th^, IV: March 18^th^ - 23^rd^ (6 days)). **A.** Daily cases and lineage identity in the Basel area cohort. Low-abundant lineages increased one week after the end of winter school vacation (March 8^th^) and were introduced by travel returners. **B.** SARS-CoV-2 lineage diversity in the Basel area, the rest of Switzerland, and neighbouring countries. Number of genomes per week are represented in the inner margin of the time wheel. Number of lineages (L) per country counted from onset of the epidemic in each country until March 23^rd^ 2020. Link to the baselayer: https://ec.europa.eu/eurostat/web/gisco/geodata/reference-data/administrative-units-statistical-units/nuts.

Lineage B.1 dominated the cases during the initial phase of the outbreak (**Fig 2A and S1 File**), with 83.6% (N = 391) of sequenced samples belonging to B.1 (**Table 3**), which was first observed in Basel on March 2^nd^. The first patient diagnosed in Basel, on February 26^th^, had a virus belonging to lineage B.1.1. This lineage was seen sporadically through the outbreak with a daily maximum of six sequenced cases on March 16^th^. Lineage B.1 is associated with the Italian outbreak [14], yet both B.1.1 (35.7%) and B.1 (51.0%) were the most prevalent lineages in Italy during this timespan (**Fig 2B**). From March 13^th^, rarer lineages were seen in the Basel area, such as B.1.1.6, a lineage that is associated with an Austrian origin [14]. Only two cases from the A lineage and sub-lineages were sequenced in the Basel area cohort (**Table 3**).

**Table 3:** Number of cases harbouring the S-D614G mutation in spike protein encoding gene in each phylogenetic lineage (PANGOLIN definition ver. May 19) and total count, in Basel area cohort by March 23rd 2020.

Phylogenetic lineage	Number of samples S^G614^ (derived)	Number of samples S^D614^ (ancestral)	Total counts
A.2	0	1	1
A.5	0	1	1
B	2	6	8
B.1	391	0	391
B.1.1	36	0	36
B.1.1.1	1	0	1
B.1.1.10	2	0	2
B.1.1.6	1	0	1
B.1.5	12	0	12
B.1.8	3	0	3
B.10	0	1	1
B.2	0	8	8
B.2.1	0	2	2
B.3	0	1	1
Sum	**448**	**20**	**468**

### Lineage diversity in Basel area, rest of Switzerland, and neighbouring countries

France was the first country in Europe that had confirmed COVID-19 cases, on January 24^th^, followed by Germany on January 27^th^, Italy on January 31^st^, and some weeks later Austria and Switzerland on February 25^th^. Comparison of lineages found in neighbouring countries over our study period shows that lineage identity and proportions vary across space and time (**Fig 2B**). Lineage B.1 dominates in France and Switzerland, whereas Italy is dominated by lineage B.1.1, and Austria exhibits a diversity of abundant lineages (**Fig 2B**). The sample proportions from Basel area residents (Basel-City and Basel-Country; 506 genomes) mostly mirror the lineage proportions of the rest of Switzerland (**Fig 2B**), partly as they contribute 77% of all available sequencing data for Switzerland within this timeframe (GISAID database as of June 22^nd^ [19,20]). Considering the time period from onset of the epidemic in each country until March 23^rd^ 2020, Switzerland (77.1%) had a similarly large proportion of B.1 lineage to France (90.0%). Viral diversity can be described based on abundance of lineages (retrieved from GISAID) using a range of diversity indices (S2 Table). Simpson diversity, which accounts for differences in sample abundance between countries, was highest in Germany (4.1) and Austria (3.7), followed by Italy (2.5); it was lowest in Switzerland (1.6) and France (1.4).

### Basel samples in global phylogenetic context

**Fig 3:**
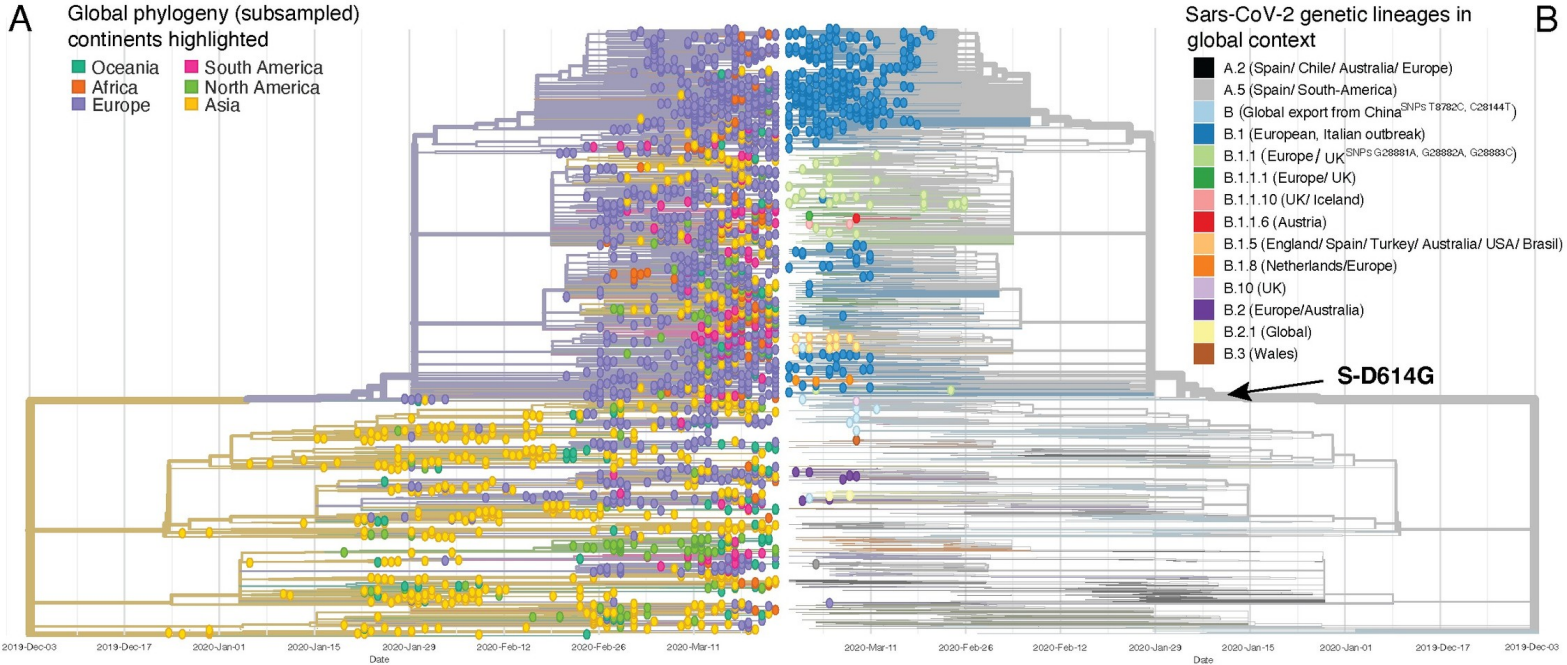
SARS-CoV-2 phylogeny of Basel area samples and genetic lineages (PANGOLIN) in a global context. **A**. Time tree of SARS-CoV-2 genomes from the Basel area cohort as well as subsampled global genomes (30 genomes per country and month), coloured by continent of origin. Amino acid mutations at internal nodes representing clade defining mutations are shown. **B.** Mirrored time tree coloured by genetic lineages sensu PANGOLIN v.May19 (https://github.com/cov-lineages/). Each tip with a circle represents a genome from the Basel area cohort, branches without circled tips represent global genomes, showing the global context of the Basel genomes.

In order to better contextualize our findings, we analysed our virus genomes phylogenetically with a subset of global publicly available sequences (**Fig 3**). While SARS-CoV-2 spread shows some geographical signal, phylogenetic lineages do not exclusively correspond to continents (**Fig 3A and S1 File**), illustrating the degree of global interconnectivity and speed of spreading. The phylogenetic lineages recorded in the Basel area are distributed across the global phylogenetic tree (**Fig 3B**). Mismatch of ‘taxonomic’ assignment and phylogenetic origin of genomes assigned to B.1 can be seen, with several as yet unnamed sub-lineages apparent in the phylogeny (**Fig 3B**).

**Fig 4:**
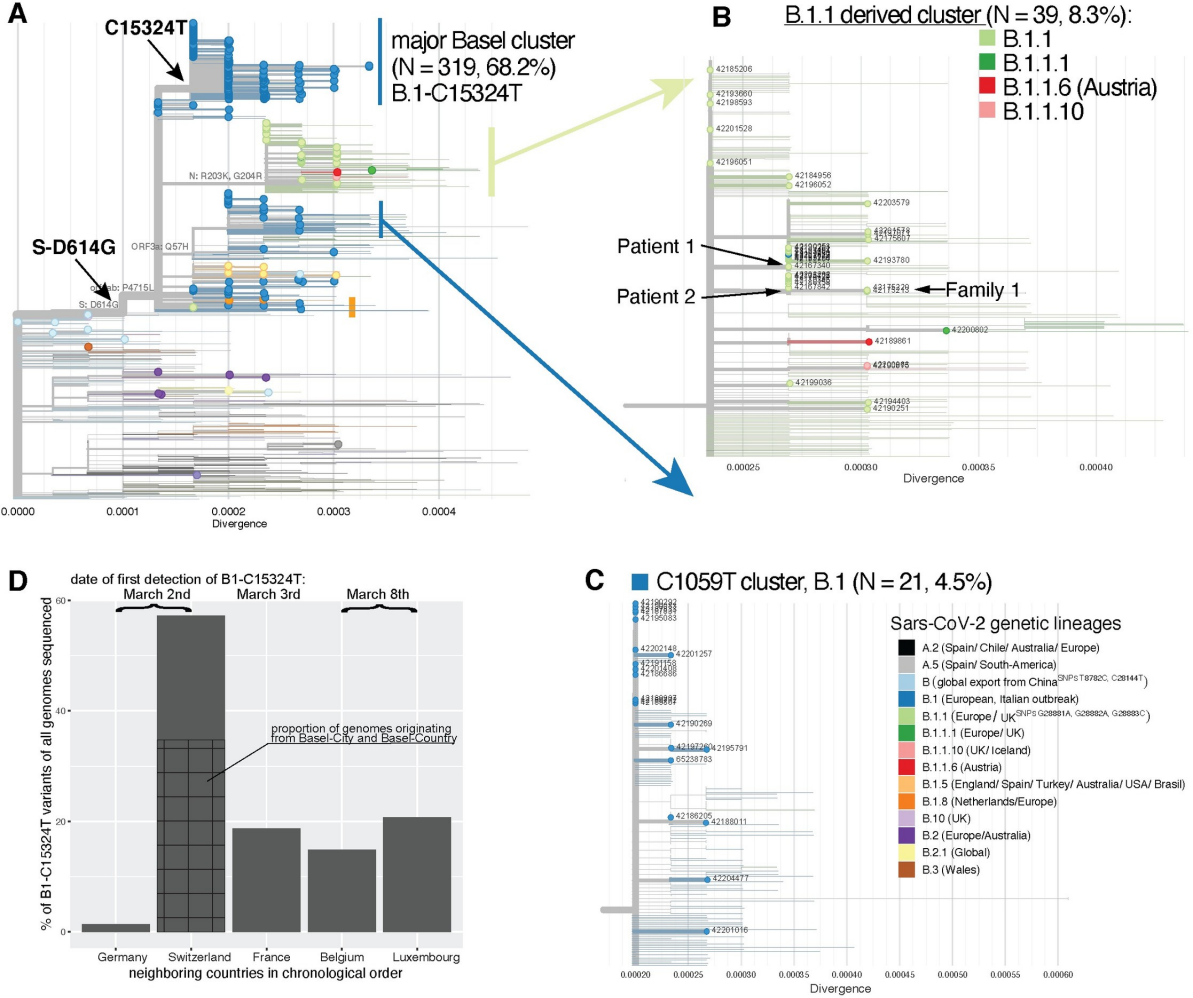
Divergence trees plotting nucleotide divergence between 468 genomes and expanded clusters of genomes in selected phylogenetic lineages. **A**. Genomes from the Basel area cohort in global context. Tree composition is identical to the time tree from Fig 3. Branches with circles at the tip represent genomes from the present study; branches without circles represent global genomes from GISAID. The major Basel cluster contains genomes with up to five mutations from its ancestral node. **B**. Detail of a mixed cluster derived from B.1.1 with seven to nine mutations to the root. A single genome assigned to lineage B.1.1.6 has an assumed origin in Austria; two genomes (B.1.1.10) most likely originate from the UK. **C**. Potential ski-holiday related cluster (C1059T) with seven samples had confirmed associations with skiing destinations. **D**. Proportion of B.1-C15324T genomes among all publicly available genomes per country from the five countries in which this variant was first registered to March 23^rd^. Note: Divergence scale can be translated to number of mutations difference to the root (Wuhan-Hu-1) by multiplication with the SARS-CoV-2 genome size (29903 bases).

We identified a major clade, within lineage B.1, B.1-C15324T, comprising 68.2% of our samples (319/468 samples with 264 (82.8%) from patients from cantons Basel-City and Basel-Landschaft; **Fig 4A**). The remaining Basel area sequences (31.8%) (**Figs 4B, 4C, S3B and S3C**) are spread throughout the phylogeny and cluster with global genomes. Introductions and features of some of the clades are analysed in the following sections and in S1 Text.

### The first identified introduction of SARS-CoV-2 to Basel

The first two positively diagnosed patients with SARS-CoV-2 in Basel and first identified cases of COVID-19, *Patient 1* and *Patient 2*, returned together from travel to Italy. In our analysis, both *Patient 1* and *Patient 2* carried viruses from the B.1.1 lineage, the second most prevalent lineage in Italy at that time (**Fig 2**). Interestingly, the two virus genomes from *Patient 1* and *Patient 2* are separated by two SNPs, suggesting two independent infections (**Fig 4B**). Moreover, we did not identify minor alleles within these samples that would hint at double infection of the patients. In this case, the epidemiological assumption that *Patient 1* and *Patient 2* were infected by the same viral source is not congruent with the phylogenetic inference.

The virus genome of *Patient 1* carries a synonymous mutation at C313T in *ORF1ab*, which is found in samples from Israel, Hungary, Japan, USA, Argentina, Greece, India, Brazil, Morocco, and Netherlands among others (nextstrain.org), all sharing an unsampled hypothetical common ancestor that emerged around February 25^th^ (CI February 23-26^th^). This SNP is also found in ten other Basel area cohort genomes: eight of these are from a family and social friends cluster unrelated to *Patient 1* that were diagnosed between March 13^th^ and March 22^nd^.

The virus genome of *Patient 2* carries a synonymous mutation at T19839C in *ORF1ab* and not C313T, and clusters together with eight identical virus genomes sampled between February 26^th^ and March 23^rd^. Two of these are from family members of *Patient 2*, who tested positive two and six days later, suggesting a family route of infection. The epidemiological data of the other six patients suggests alternative sources: one of the six returned from a Swiss ski resort three days prior to onset of symptoms. Two additional samples forming a family cluster (*Family 1*, **Fig 4B**) diagnosed on March 3^rd^, carry the T19839C plus non-synonymous mutation G28179A leading to amino acid change ORF8-G96S. One member of *Family 1* travelled with *Patients 1* and *2* to Italy and possibly got infected there with a yet different virus variant.

### Introduction of the Basel cluster

The clade B.1-C15324T, within which 68.2% (N = 319) of our Basel area cohort sequences fall, henceforth referred to as the “Basel cluster”, is characterized by synonymous SNP C15324T in *ORF1ab*. *Patient 1* and *Patient 2* are not linked to this Basel cluster. The first sample within the Basel cluster, from March 2^nd^, was from a patient residing in Central Switzerland, who was transferred to a care facility in Basel-City where six further people tested positive between March 5^th^ and March 17^th^ with identical viral genomes. The source of the first infection is unknown. The second sample within the Basel cluster was from a patient diagnosed on March 3^rd^, who had attended a religious event in Alsace, France, that took place between February 17^th^ and 21^st^. One other patient, who tested positive on March 9^th^ but had symptoms 14 days prior to testing, had an identical viral genome and had also attended this event. Several other positively tested patients, from whom we could not successfully sequence virus genomes, also attended this event.

### Description of the Basel cluster

The 319 genomes in the Basel cluster (**Fig 4A**) show a divergence of up to five SNPs up to March 23^rd^, of which 157 genomes are identical and located at the root of the clade. This infers that the common ancestor originated between February 9^th^ and February 17^th^. The clade defining SNP C15324T (GISAID emerging clades label 20A/15324T) was identified for the first time on March 2^nd^ simultaneously in the Basel area sample 42173111 and GISAID sample Germany/FrankfurtFFM7/2020, suggesting unsampled circulation of this variant in Europe from mid-February. By searching all genomes available on GISAID (N = 80,189; as of August 12^th^ 2020), filtering for genomes belonging nextstrain emerging clade 20A/15324 and sampled until March 23^rd^ (N = 2,856), we find that the C15324T mutation has subsequently been observed in other countries but remains most prevalent in Switzerland (N_GISAID_ = 57/213, 26.8%; N_GISAID+this study_ = 386/675, 57.2%; first genome 42173111 from March 2^nd^) (**Fig 4D** and S3 Table). Of the 57 Swiss genomes with this mutation sequenced also by other groups, 26 originate from the cantons Basel-City and Basel-Landschaft (**Fig 4D** and S4 Table). The mutation is also found in high proportions in genomes from France (N = 69/369, 18.7%, first from March 3^rd^ sample France/HF1870/2020), Luxembourg (N = 24/116, 20.7%, from March 8^th^ sample Luxembourg/LNS2614631/2020), and Belgium (N = 40/268, 14.9%, from March 6^th^ sample Belgium/NKR-030645/2020): these are all within a few days of the first recorded occurrence from Switzerland and Germany (**Fig 4D** and S3 Table). Subsequently, as of March 9^th^, descendants of this variant were recorded outside Europe (S4 Table) in smaller proportions than in Basel (S3 Table), which suggests dissemination from the Basel area.

### Potential ski-holiday related cluster (C1059T)

Previous reports have identified viruses in lineage B.1 carrying SNP C1059T (amino acid change ORF1a-T265I) in travel returners from ski holidays in Ischgl, Austria [9,21]. We detected 21 (4.5%) viral samples within our cohort with this feature (**Fig 4C**), divergent by up to two SNPs from the common ancestor. Samples date from March 11^th^ to March 23^rd^ with an inferred internal node age of February 21^st^ (CI: February 20^th^-February 21^st^ 2020), fitting with possible infections in Ischgl from end of February to March 13^th^ [22]. Epidemiological data confirms that five patients from that cluster had returned from Austria, three specifically from Ischgl, before testing positive. Two additional patients within this cluster returned from skiing in Swiss resorts.

### Spike protein mutation prevalent in Basel patients

The spike protein S-D614G mutation is associated with the B.1 lineage and all those derived from this (**Fig 4**). As such, it occurs in 448 of the 468 (95.7%) samples from the Basel area. The SNP responsible has not been lost once in our sub-sampled dataset, but it is not present in B.2 or B.3 or other sister lineages to B.1 (**Table 3**). Among our samples, we found no significant difference in viral loads between patients with and without the S-D614G mutation (z = -0.881, p = 0.38). However, our cohort is biased to samples with higher viral loads, as these were successfully sequenced.

## Discussion

We reconstruct the early events of SARS-CoV-2 in Basel and the surrounding area, from a phylogenetic perspective, focusing on introduction and spread. We present COVGAP, a new combination of existing tools to effectively mine SARS-CoV-2 genomes from Illumina data. Unlike other currently available tools [23], COVGAP shows higher sensitivity levels in SNP calling from raw reads (100%), failing only in ambiguously mapped deletions and insertions. In such cases, it adopts a coverage-conservative approach, needed to reliably call variants in real world scenarios.

The majority of genome variants in Basel are similar to those from France, Italy, and Germany. We identified 13 SARS-CoV-2 lineages in our samples, with the Basel outbreak powered by the European B.1 lineage. In particular, the B.1-C15324T lineage variant dominated the early phase of the local spread causing approximately 70% of Basel cases. Compared to Victoria, Australia [5], the UK [24], or Austria (this study) the diversity seen arriving in Switzerland and Basel as determined using Simpson diversity is more limited, perhaps reflecting European rather than intercontinental connections. This diversity measure is easily produced and can be used by public health institutions to monitor the effect of travel restrictions on viral introductions.

The Basel cluster virus variant 20A/C15324T was first detected in Europe on March 2^nd^ in Switzerland and Germany simultaneously. We locate its geographic origin to our tri-national region between February 9^th^ and February 17^th^. The first recognized case in the large Basel cluster goes back to a patient in a care facility, in which several more infections occurred. Our epidemiologically-informed phylogenetic analysis indicates that the Basel cluster represents a larger transmission chain that was unchecked and spread effectively among unrelated people throughout Basel and eventually outside Europe.

The beginning of the COVID-19 outbreak described here coincided with the winter school holidays in Basel, from February 22^nd^ to March 8^th^ 2020. This holiday provided the opportunity for the first identified SARS-CoV-2 introductions to Basel through two jointly returned travellers from Italy, notably each with different viral variants. During this time, many residents also travelled to ski resorts. Viral introductions from ski resorts are known from contract tracing data to have affected Germany [25], Denmark [21], Iceland [9], France, Spain, and UK [26], with Ischgl, Austria described as a source of many cases. Our data supports the finding that ski resorts in Austria and Switzerland served as dissemination hotspots. Overall, however travel returners did not drive the outbreak in Basel evidenced by the low diversity of variants and low proportion of such variants in our sample. This is possibly due to the heightened attention and restrictions, which were already imposed on travellers (recommended quarantine) but not on social events. A second likely source represents the many workers travelling daily across borders from France and Germany, particularly from heavily affected areas such as Alsace [18]. As the B.1 and B.1.1 lineages were dominant in France and Germany, these workers may have provided the sources of some cases and transmissions.

The timing of the epidemic in Basel coincided with three major events. Firstly, a religious event from February 17^th^ to 21^st^ in Alsace that was described as a super-spreading event in France [27]. We confirmed that the virus genomes of two patients known to have attended are indeed situated at the root of the clade that constitutes the Basel outbreak, and further patients that attended the event also tested positive. Other major events occured in Basel during the same time period but epidemiological links for those are lacking, as relevant questions were not posed in the patient questionnaire. One is the carnival in the Basel area that is celebrated over several weeks from early January, with numerous events, and thousands of active participants. The culmination is the UNESCO world-heritage ‘Basler Fasnacht’, scheduled 2020 for March 2^nd^-4^th^_,_ but cancelled due to COVID-19. Notably, active participants practice the piccolo and drums over the preceding weeks in closed rooms as a preparation for their performance during the carnival, and unofficial events during Fasnacht are likely to have taken place. Additionally, three international soccer events took place at the St. Jakob Stadium, Basel, on February 15^th^ (with 20,675 spectators), 23^rd^ (20,265 spectators), and 27^th^ (14,428 spectators).

The availability of epidemiological data and integration to aid in the interpretation of phylogenetic clades underlines the validity of instrumentalising those tools for improving the understanding of SARS-CoV-2 outbreak dynamics. Utilizing the clades as the backbone for targeted epidemiological analysis of specific cases helped to grasp how mass gatherings, travel returners, and care facilities may influence an outbreak within a city. The epidemiological data that was collected for the Federal Office of Public Health (FOPH), as requested by law, helped tremendously to verify travel related links; however it was not designed to obtain data on local super-spreading events such as attendance to soccer games, visiting clubs, restaurants, bars, and concerts and future versions could be improved. The classical epidemiological context is very important to further explain molecular epidemiological links especially in an as yet not very diversified virus.

A limitation of the current study is that we are likely to have missed some cases as not all symptomatic people were advised to be tested, especially children younger than 18 years old. Nevertheless, our cohort represents a very high sequencing density per detected case for a city (468 genomes from 746 PCR-confirmed cases in Basel area (62.7%) and from 10,680 PCR-confirmed cases nationwide (4.4%)) for this early phase of the pandemic [28].

In conclusion, the start of the outbreak of SARS-CoV-2 in the Basel area was characterized by a dominant variant, B.1-C15324T, which we infer to have arisen in mid-February in our tri-national region. Large gatherings (potential super spreading events) appear to have had profound effects on outbreak dynamics. Our analysis shows the potential of molecular epidemiology to support classical contact tracing, currently or retrospectively, in order to evaluate and improve measures to contain epidemics like COVID-19.

## Materials and methods

### Ethics statement

The study was conducted according to good laboratory practice and in accordance with the Declaration of Helsinki and national and institutional standards. The study was assessed and approved by the local ethical committee (Ethikkommission Nordwest und Zentralschweiz, www.eknz.ch; ID number: 2020–00769). No signature was required according to the ethical assessment. All data from study participants (pediatric and adult patients) were reported in an anonymous fashion. The clinical trial accession number is NCT04351503 (clinicaltrials.gov).

### Patients, samples, and diagnosis

Respiratory samples from the University Hospital Basel and the University Children’s Hospital Basel (UKBB) patients were tested for SARS-CoV-2: from January 23^rd^ 2020 testing was based on current case definitions from the Federal Office of Public Health (FOPH); from 27^th^ February additionally, all respiratory samples negative for other respiratory pathogens were tested. Patient samples which tested positive for SARS-CoV-2 [29,30] up to and including March 23^rd^ were considered eligible for the present study. In total 6,943 diagnostic tests were performed during the study period. The 746 positively tested cases came predominantly from the administrative unit of Basel-City, Riehen, and Bettingen (418, 58%), while the remaining patients were from Basel-Landschaft and neighbouring cantons and countries.

For diagnosis, swabs from the naso- and oropharyngeal sites (NOPS) were taken, and combined into one universal transport medium tube (UTM, Copan). Total nucleic acids (TNAs) were extracted using the MagNA Pure 96 system and the DNA and viral RNA small volume kit (Roche Diagnostics, Rotkreuz, Switzerland) or using the Abbott m2000 Realtime System and the Abbott sample preparation system reagent kit (Abbott, Baar, Switzerland). Aliquots of extractions were sent for diagnosis to Charité, Berlin, Germany from January 23^rd^ - 29^th^, and to Geneva to the National Reference Centre (NAVI) in Switzerland from January 29^th^. In-house analysis started February 27^th^ as part of the hospital routine diagnostics as previously described [30].

### Whole genome sequencing (WGS)

SARS-CoV-2 genomes were amplified following the amplicon sequencing strategy of the ARTIC protocol (https://artic.network/ncov-2019) with V.1 or V.3 primers [31]. In detail, real-time reverse transcriptase (RT) reactions were run to a total volume of 10μl extracted total nucleic acid. After some optimization, PCR used 25 cycles for samples with a diagnostic cycle threshold (C_t_) value lower than 21 (viral loads higher than 8.2 log10 Geq/ml); 40 cycles for all other samples (lower viral load samples) and repeats. Purified amplicons were converted into Illumina libraries with Nextera Flex DNA library prep kit (Illumina) automated on a Hamilton STAR robot, using 5ng input DNA. 96 libraries were multiplexed and sequenced paired-end 150 nucleotides on an Illumina NextSeq 500 instrument.

### Consensus sequence generation and detection of mutations

After demultiplexing using bcl2fastq software version v.2.17 (Illumina), COVGAP (COVid-19 Genome Analysis Pipeline) was used (S4 Fig). This incorporates: quality filtering using trimmomatic software version v.0.38 [32] to remove Illumina adaptors and PCR primer sequences from read ends; removal of reads smaller than 127 bases, and removal of reads with a phred score under 20 (calculated across a 4-base sliding window). Quality filtered reads were mapped to the Wuhan-Hu-1 reference MN908947.3 [33] using the BWA aligner [34]. Reads flagged as mapping to the reference were retained [35], and are deposited under project PRJEB39887. SNPs and indels with respect to the reference sequence were called using pilon version 1.23 [36]. Pilon summary metrics ‘alternative allele fraction’ (AF) and ‘depth of valid reads in pileup’ (DP) were used to identify major and minor alleles across all bases, which is not implemented in pilon itself. Major alleles were called if supported by 70% of the reads covering the variant locus (AF) for any locus with a minimum of 50x coverage (DP). Variants were applied to the reference to produce a consensus sequence; any base position with less than 50x coverage was masked with ambiguous characters (Ns) using BCFTools version 1.10.2 [37]. Consensus sequences were accepted for further analysis when containing up to 10% Ns. Summary statistics, logs, coverage plots, and genome stack plots were generated using R version 3.6.0 and packages Gviz v1.30 [38], Sushi v1.23 [39], seqinr v3.6.1 [40], and ggplot2 v3.11 [41]. COVGAP also provides per genome quality control visual outputs (S5 Fig) and is available at https://github.com/appliedmicrobiologyresearch.

Quality control statistics such as the relationship between C_t_-value and number of mapped reads and coverage are presented in S5 Fig. In general, we observed a negative trend linking C_t_ values and percentage of ambiguous bases (Ns) being called as a result of low coverage. Sequences passing the quality filter (n = 533) showed a lower C_t_ value (median: 22.4±5.14) than the ones that failed (n = 156; median: 35.75.9±5.75).

### COVGAP validation

We used a set of 15 randomly *in silico* mutated SARS-CoV-2 mock genomes for the validation of the specificity (identification of true negatives) and accuracy (identification of true negatives and true positives) of COVGAP. Additionally, the genome MT339040, which harbours an 81 nucleotide deletion in the ORF7a gene and a further seven SNPs relative to the reference [42] was used. Together, the mock genomes possess 38 mutations including 30 SNPs, six deletions and two insertions across the reference genome MN908947.3. The genomes were then shredded to artificial paired-end 150 nucleotide reads using SAMtools wgsim [37] and processed by COVGAP. For validation purposes, original mock genomes and the COVGAP generated genomes from the shredded reads were aligned using Seaview v4.6 [43] and clustalw [44]. A phylogeny was built using PhyML within Seaview with default parameters.

### Phylogenetic lineage assignment of Basel samples

To assess the phylogenetic diversity of SARS-CoV-2 samples during the early phase of the pandemic we inferred the lineage assignment for each consensus sequence derived from the COVGAP pipeline using PANGOLIN ver. May 19th (Phylogenetic Assignment of Named Global Outbreak LINeages) [14] available at github.com/hCoV-2019/pangolin. Details on lineage summaries, describing which countries lineages have been reported from and where transmission events have been recorded, can be found at https://github.com/hCoV-2019/lineages. Lineage assignments were used to aid visualization of phylogenetic diversity in Basel in a global context. For global sequences we used the PANGOLIN lineage assignments as provided by GISAID (https://www.gisaid.org/; [19,20]) (details next section), which were used for plotting purposes on phylogenetic trees.

To visualise the lineage diversity in Switzerland, the Basel area, and neighbouring European countries (Austria, France, Germany, and Italy) during the first 27 days of the pandemic in Basel, we visualized relative abundances of lineages using all high-quality, on GISAID (downloaded June 22^nd^ 2020) available consensus sequences for the time until March 23^rd^ from Austria (N = 188), France (N = 230), Germany (N = 133), and Italy (N = 98). For Switzerland (N = 673), we combined our sequences (N = 468) with other sequence data from Switzerland published on GISAID as of June 22^nd^ 2020. To visualize the diversity for the Basel area we included sequnces from Basel-City (including Bettingen and Riehen) and Basel-Country (N = 506). To infer diversity, we calculated Simpson diversity (inverse Simpson concentration) for Switzerland, Germany, Italy, Austria, and France from onset of the pandemic in each country until March 23^rd^ 2020, as implemented in the SpadeR package v.0.0.1 [45–47], which controls for lineage abundance differences between the countries, which is dependent on available sequence data, and which ranges from 0 (no diversity) to indefinite (large diversity).

### Analysing Basel SARS-CoV-2 genomes in global phylogenetic context

High-quality and full-length consensus sequences and corresponding metadata (sample ID, date of sample, geographic location of sampling, PANGOLIN lineage) from Swiss [48] and global viruses were downloaded from GISAID on June 22^nd^ 2020, making 49,284 individual genome sequences. 43,252 sequences were retained after filtering for genomes with under 10% ambiguous characters (Ns) (author Genivaldo Gueiros Z. Silva) [49]. Metadata and consensus sequences of the Basel samples and global data from GISAID were combined for further joint analysis, which were performed using custom R scripts and the nextstrain command line interface analysis pipeline v.2.0.0 (nextstrain.org) and augur v.8.0.0 [50].

Dates in our study samples correspond to date of sampling. Sequences were filtered by date from December 1^st^ 2019 to March 23^rd^ 2020 using an R custom script in R version 4.0.0 [51] and packages tidyr ver.1.1.0 [52], dplyr ver. 1.0.0. [53], and readr ver. 1.3.1. [54]: 15,973 consensus sequences, including the Basel area sequences, remained. These time-filtered sequences were sub-sampled by geographic location to 30 sequences per country and month. Non-human derived viruses as well as sequences with other ambiguous characters (Us), as well as those from cruise ships, and duplicated sequences defined by the nextstrain team as of June 24^th^ (https://github.com/nextstrain/ncov/) were excluded using *augur filter* [50] resulting in 2,485 sequences for the final phylogenetic analysis dataset.

Consensus sequences were aligned to the NCBI Refseq sequence Wuhan-Hu-1 reference MN908947.3 using mafft v7.467 with method FFT-NS-fragment [55] and options—reorder—keeplength—mapout—kimura 1—addfragments—auto. The resulting alignment was end-trimmed to remove low-quality bases (bases 1–55; 29804–29903). We masked homoplasic sites (**S6 Table**) that have no phylogenetic signal [56] (deposited at https://github.com/W-L/ProblematicSites_SARS-CoV2). Please note, that this list is under constant development as number and diversity of sequence data evolves; we retrieved the data on June 19^th^ 2020. Masking was done using *augur mask*.

The resulting alignment was analysed in IQ-TREE 2 [57] for tree inference using *augur tree* with substitution model GTR+G. The tree in Newick format was then subjected, together with the date information of each genome and the initial sequence alignment, to an estimation of the evolutionary rate by a regression of the divergence (number of mutations) against the sampling date using TreeTime [58] implemented in *augur refine*. Genomes or branches that deviated more than four interquartile ranges from the root to the tip versus the time tree were removed as likely outliers. The resulting time-calibrated and divergence trees were re-rooted to MN908947.3 and MT291826.1, the first official cases and published genomes of SARS-CoV-2 from Wuhan, China.

Ancestral trait reconstruction of each patient’s viral genome was done for region (continent) and country as well as region and country of exposure using *augur* traits with a sampling bias correction of 2.5. Internal nodes and tips (actual genomes) were annotated regarding their nucleotide and amino acid changes in relation to the reference using *augur ancestral* and *augur translate*, respectively. All data were exported as json file (**S1 File**) using *augur export v2* to be visualized in *auspice* v2 [50].

Identified clades of interest were further inspected for existing epidemiological links using data collected by the University Hospital Basel.

### Identifying genomes belonging to GISAID emerging clade A20/15324T

To identify a possible geographic origin of the synonymous C15324T mutation in *ORF1ab*, we performed a search on all available GISAID genomes as of August 12^th^ 2020. We downloaded all high quality and complete genomes that were assigned do GISAID legacy clade G (corresponds to clade 20A) and PANGOLIN lineage B.1 (all three are mostly congruent [59]) with a collection date between December 2019 and March 23^rd^ 2020 (N = 2,856). We used Nextclade version 0.3.5 (https://clades.nextstrain.org) to infer genomic mutations and filtered for sequences that contained C15324T. This procedure allowed avoidance of homoplasic mutations at this site. Further, we downloaded metadata for all high quality and complete genomes (as of August 12^th^ 2020) irrespective of clade to calculate summary statistics of number of genomes sequenced per country.

### Identification of S-gene D614G mutation in Basel sequences

We screened the early phase Basel sequences for the mutation at nucleotide position 23,403 based on the alignment to the *Wuhan-Hu-1* reference sequence MN908947.3. Viral load (C_t-_value) of patients that carried lineages with a mutated S-D614G gene (N = 274) were compared to patients that carried the ancestral allele (N = 12) using a Mann-Whitney U test.

## Supporting information

S1 Fig Age distribution by sex for the time period between February 24th and March 23rd for all tests, positive tests, and for patient isolates from which whole genomes were generated. Solid lines for females, dashed lines for males.
(PDF)

S2 Fig COVGAP identifies all SNPs in the mock genomes. **A**. Levenshtein distance between the mutations in the input mock genomes (y axis) and in the genomes recovered by COVGAP (x axis); the marginal plots show the frequency of presence / detection across samples. Only two deletions (5845, 16281) and one insertion (16145) were not detected. **B**. Phylogeny of input and COVGAP-derived consensus output genomes, showing that all SNPs were identified.
(PDF)

S3 Fig Divergence tree and zoom into additional sequence clusters, which did not result in large community spread. **A**. Isolates from Basel area cohort in global context. **B**. A small clade assigned to B.1.5 consists of two clusters with an accumulation of samples from Basel. **C**. Cluster within lineage B.1.8 with two Basel samples without known epidemiological link. **D**. Two family cluster within lineage B.2 that did not spread further into the Basel community.
(PDF)

S4 Fig The COVGAP pipeline. The steps shown ensure the calculation of high quality consensus sequences. Particularly, information on read coverage is retained and used both in the variant calling procedure and in the draft of the consensus independently from the called variants. Finally, the quality of the genome from each sample is scored by %Ns, which determines whether the produced sequence is retained or discarded. Support for the creation of this schematic figure was provided by BioRender.com.
(PDF)

S5 Fig Representative diagnostic output from COVGAP. This output generated per sample, indicates (from top to bottom): **A**. the coverage–not represented if over 1000x; **B**. which variants were detected in which position of the genome, and their corresponding annotation; **C**. low coverage regions (under 50x); **D**. genome annotation; and **E**. genome size markers as reference. Of note, a report generated in parallel provides further information on the variants, including which amino acids are affected by the variant.
(PDF)

S6 Fig COVGAP evaluation of sequencing quality parameters. Of the original 746 samples, 689 successfully sequenced. Number of mapped reads across all SARS-CoV-2 positive samples successfully sequenced from 26th of February till 23th of March: (n = 689), of which 533 passed the quality filter, and 156 failed. 468 of the samples passing the quality filters were matching the cohort eligibility criteria and therefore were further described in the present study. **A.** Number of mapped reads against Ct values from diagnostic tests; **B.** Number of mapped reads against percentage of Ns in the consensus.
(PDF)

S1 Table Counts and description of the in silico mutated genome community used for COVGAP validation. Each observation consists of the genome position multiplied by the number of samples in which it appears.
(PDF)

S2 Table Diversity indices for SARS-CoV-2 lineages in Switzerland and neighbouring countries for the time period of first detected case until March 23rd, 2020. (PDF)

S3 Table List of countries that recorded genomes with mutation C15324T in B.1 background and number of total genomes sequenced until March 23rd 2020. (PDF)

S4 Table GISAID identifiers and dates of sampling for all sequences that belong to emerging clade 20A/15324T with a collection date until March 23rd, 2020 (N = 279). (PDF)

S5 Table GISAID author acknwoledment file. (TSV)

S6 Table Nucleotide position in relation to the Wuhan-Hu1 reference sequence that were masked for phylogenetic inferences, due to homoplasies. Inferred by contributors to https://github.com/W-L/ProblematicSites_SARS-CoV2.
(PDF)

S1 File Json tree file. (JSON)

S1 Text Supplementary results. (PDF)
