# Supplementary material for: SARS-CoV-2 outbreak in a tri-national urban area is dominated by a B.1 lineage variant linked to a mass gathering event
Source: PLoS Pathog. 2021 Mar 19;17(3):e1009374. doi: 10.1371/journal.ppat.1009374 (PMC8011817; doi:10.1371/journal.ppat.1009374)
Supplement: S1 Fig — Solid lines for females, dashed lines for males. (PDF) [file ppat.1009374.s001.pdf]

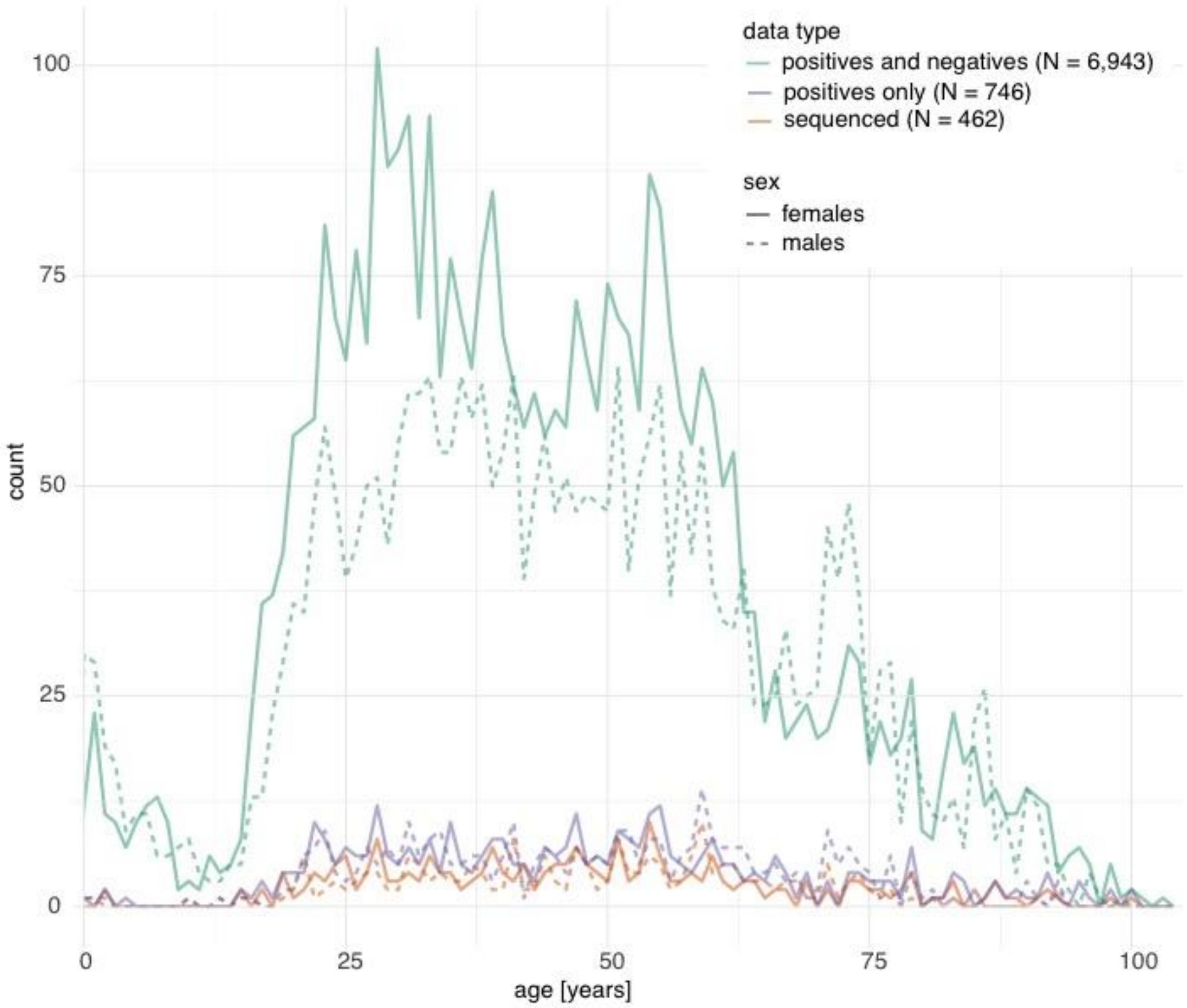

Figure S1. Age distribution by sex for the time period between February 24th and March 23rd for all tests, positive tests, and for patient isolates from which whole genomes were generated. Solid lines for females, dashed lines for males.
