# Supplementary material for: SARS-CoV-2 outbreak in a tri-national urban area is dominated by a B.1 lineage variant linked to a mass gathering event
Source: PLoS Pathog. 2021 Mar 19;17(3):e1009374. doi: 10.1371/journal.ppat.1009374 (PMC8011817; doi:10.1371/journal.ppat.1009374)
Supplement: S2 Fig — A. Levenshtein distance between the mutations in the input mock genomes (y axis) and in the genomes recovered by COVGAP (x axis); the marginal plots show the frequency of presence / detection across samples. Only two deletions (5845, 16281) and one insertion (16145) were not detected. B. Phylogeny of input and COVGAP-derived consensus output genomes, showing that all SNPs were identified. (PDF) [file ppat.1009374.s002.pdf]

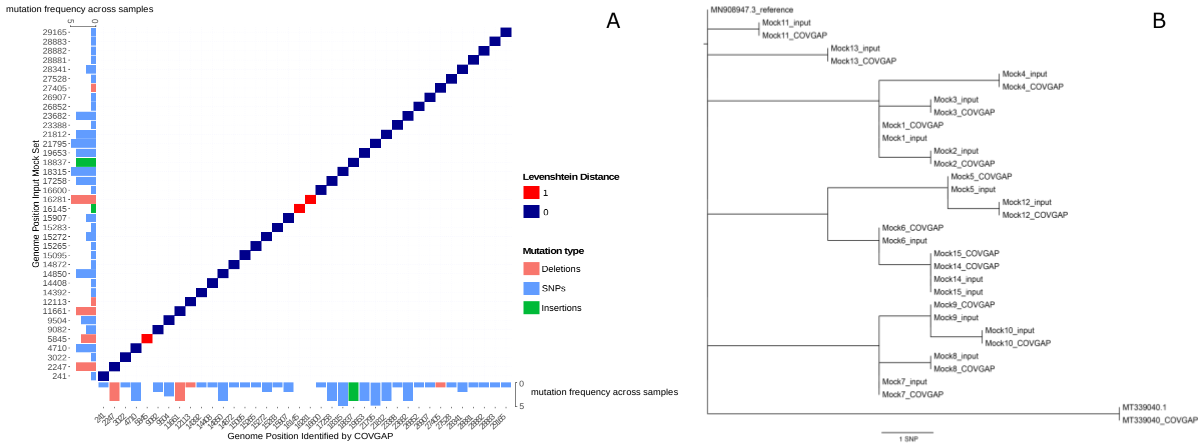

Figure S2. COVGAP identifies all SNPs in the mock genomes. A. Levenshtein distance between the mutations in the input mock genomes (y axis) and in the genomes recovered by COVGAP (x axis); the marginal plots show the frequency of presence / detection across samples. Only two deletions (5845, 16281) and one insertion (16145) were not detected. **B**. Phylogeny of input and COVGAP-derived consensus output genomes, showing that all SNPs were identified.
