# Supplementary material for: SARS-CoV-2 outbreak in a tri-national urban area is dominated by a B.1 lineage variant linked to a mass gathering event
Source: PLoS Pathog. 2021 Mar 19;17(3):e1009374. doi: 10.1371/journal.ppat.1009374 (PMC8011817; doi:10.1371/journal.ppat.1009374)
Supplement: S3 Fig — A. Isolates from Basel area cohort in global context. B. A small clade assigned to B.1.5 consists of two clusters with an accumulation of samples from Basel. C. Cluster within lineage B.1.8 with two Basel samples without known epidemiological link. D. Two family cluster within lineage B.2 that did not spread further into the Basel community. (PDF) [file ppat.1009374.s003.pdf]

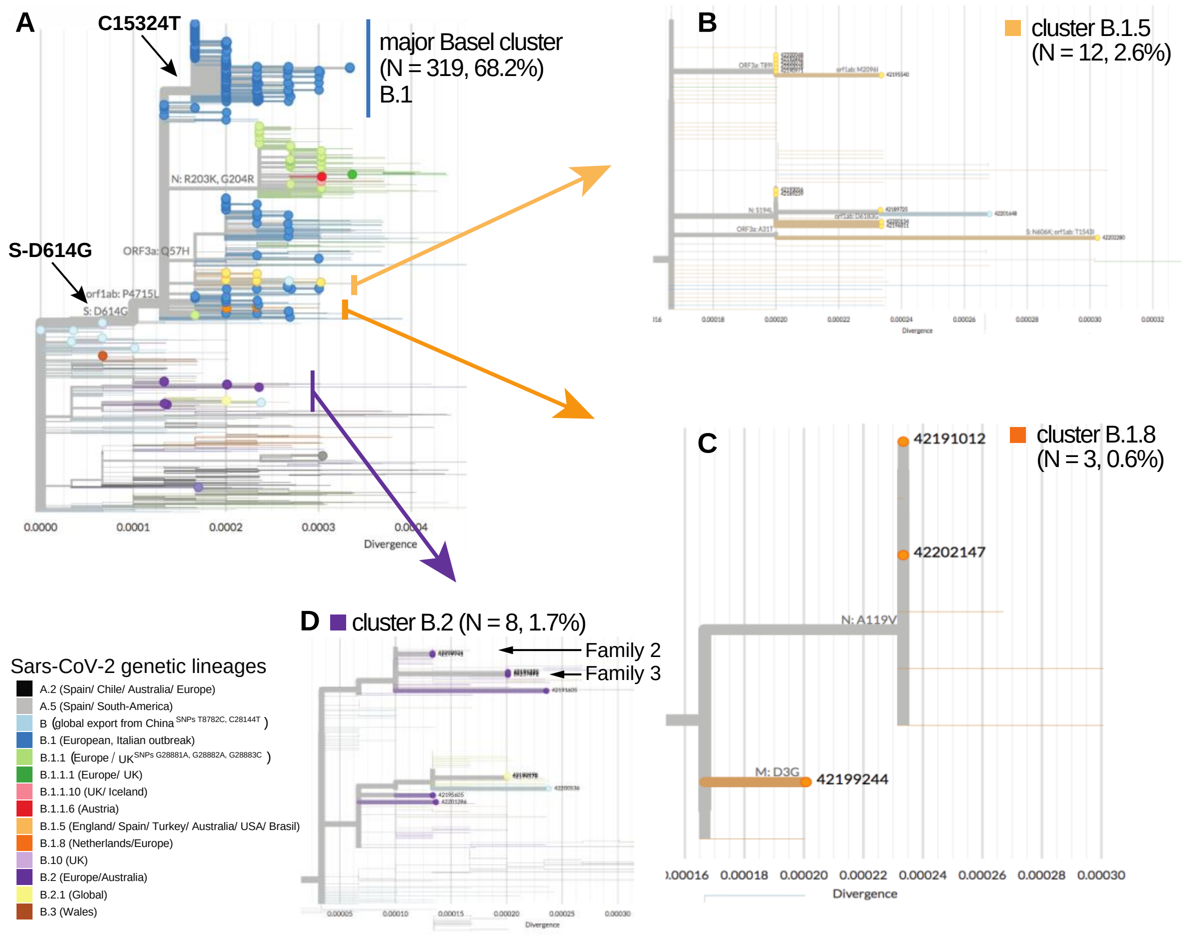

Figure S3. Divergence tree and zoom into additional sequence clusters, which did not result in large community spread. A. Isolates from Basel area cohort in global context. **B**. A small clade assigned to B.1.5 consists of two clusters with an accumulation of samples from Basel. **C**. Cluster within lineage B.1.8 with two Basel samples without known epidemiological link. **D**. Two family cluster within lineage B.2 that did not spread further into the Basel community.
