# Supplementary material for: SARS-CoV-2 outbreak in a tri-national urban area is dominated by a B.1 lineage variant linked to a mass gathering event
Source: PLoS Pathog. 2021 Mar 19;17(3):e1009374. doi: 10.1371/journal.ppat.1009374 (PMC8011817; doi:10.1371/journal.ppat.1009374)
Supplement: S4 Fig — The steps shown ensure the calculation of high quality consensus sequences. Particularly, information on read coverage is retained and used both in the variant calling procedure and in the draft of the consensus independently from the called variants. Finally, the quality of the genome from each sample is scored by %Ns, which determines whether the produced sequence is retained or discarded. Support for the creation of this schematic figure was provided by BioRender.com. (PDF) [file ppat.1009374.s004.pdf]

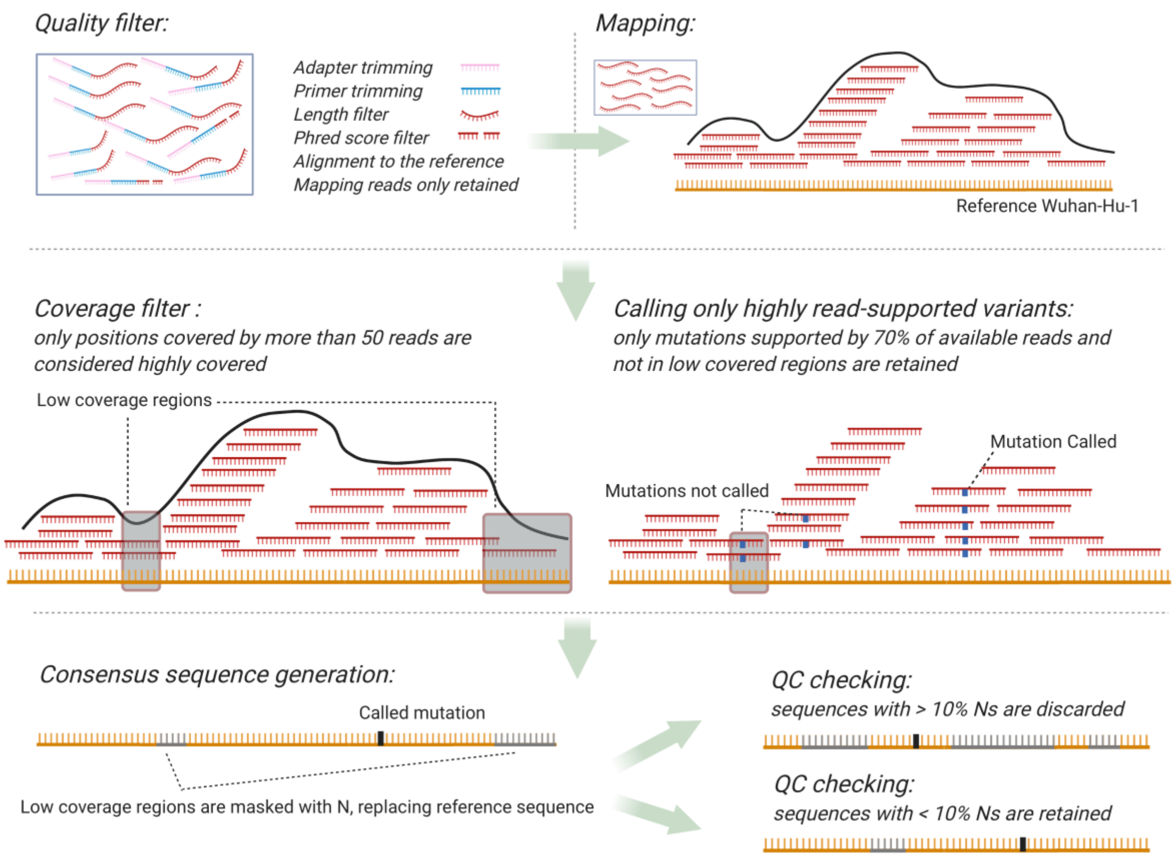

Figure S4. The COVGAP pipeline. The steps shown ensure the calculation of high quality consensus sequences. Particularly, information on read coverage is retained and used both in the variant calling procedure and in the draft of the consensus independently from the called variants. Finally, the quality of the genome from each sample is scored by %Ns, which determines whether the produced sequence is retained or discarded. Support for the creation of this schematic figure was provided by BioRender.com.
