# Supplementary material for: SARS-CoV-2 outbreak in a tri-national urban area is dominated by a B.1 lineage variant linked to a mass gathering event
Source: PLoS Pathog. 2021 Mar 19;17(3):e1009374. doi: 10.1371/journal.ppat.1009374 (PMC8011817; doi:10.1371/journal.ppat.1009374)
Supplement: S5 Fig — This output generated per sample, indicates (from top to bottom): A. the coverage–not represented if over 1000x; B. which variants were detected in which position of the genome, and their corresponding annotation; C. low coverage regions (under 50x); D. genome annotation; and E. genome size markers as reference. Of note, a report generated in parallel provides further information on the variants, including which amino acids are affected by the variant. (PDF) [file ppat.1009374.s005.pdf]

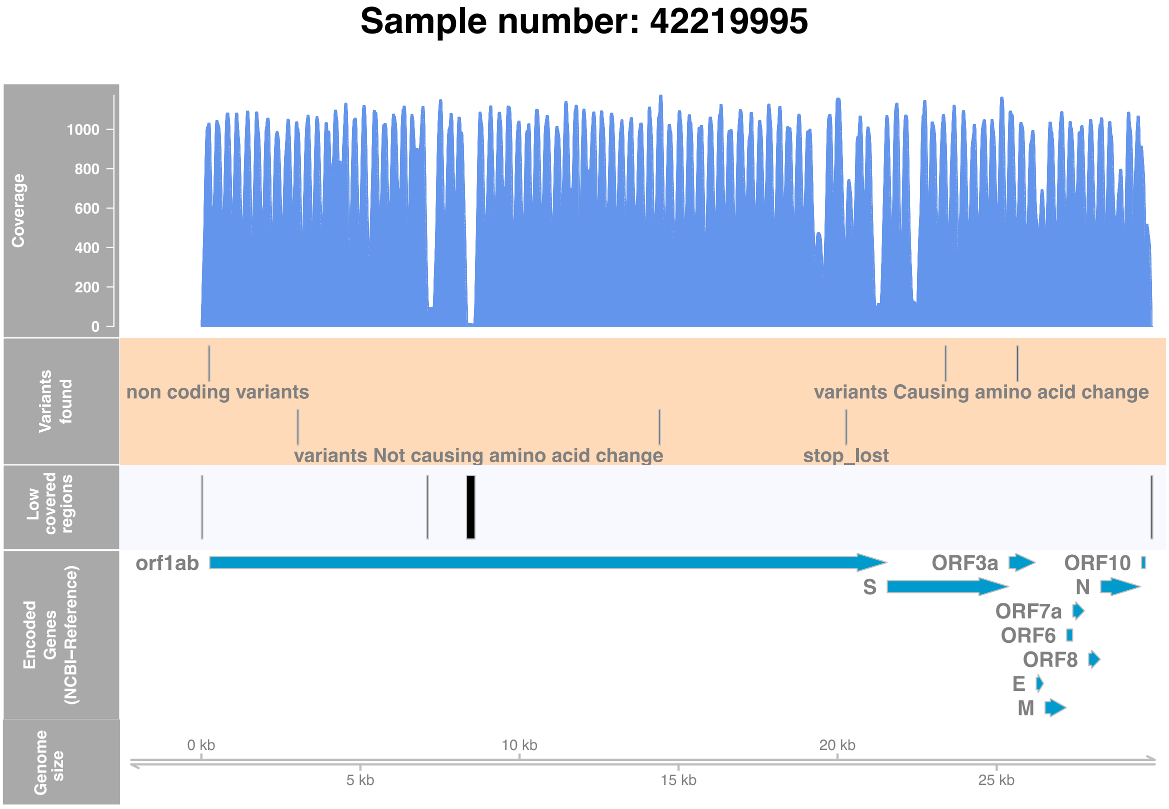

Figure S5. Representative diagnostic output from COVGAP. This output generated per sample, indicates (from top to bottom): **A**. the coverage –not represented if over 1000x; **B**. which variants were detected in which position of the genome, and their corresponding annotation; **C**. low coverage regions (under 50x); **D**. genome annotation; and **E**. genome size markers as reference. Of note, a report generated in parallel provides further information on the variants, including which amino acids are affected by the variant.
