# Supplementary material for: SARS-CoV-2 outbreak in a tri-national urban area is dominated by a B.1 lineage variant linked to a mass gathering event
Source: PLoS Pathog. 2021 Mar 19;17(3):e1009374. doi: 10.1371/journal.ppat.1009374 (PMC8011817; doi:10.1371/journal.ppat.1009374)
Supplement: S6 Fig — Of the original 746 samples, 689 successfully sequenced. Number of mapped reads across all SARS-CoV-2 positive samples successfully sequenced from 26th of February till 23th of March: (n = 689), of which 533 passed the quality filter, and 156 failed. 468 of the samples passing the quality filters were matching the cohort eligibility criteria and therefore were further described in the present study. A. Number of mapped reads against Ct values from diagnostic tests; B. Number of mapped reads against percentage of Ns in the consensus. (PDF) [file ppat.1009374.s006.pdf]

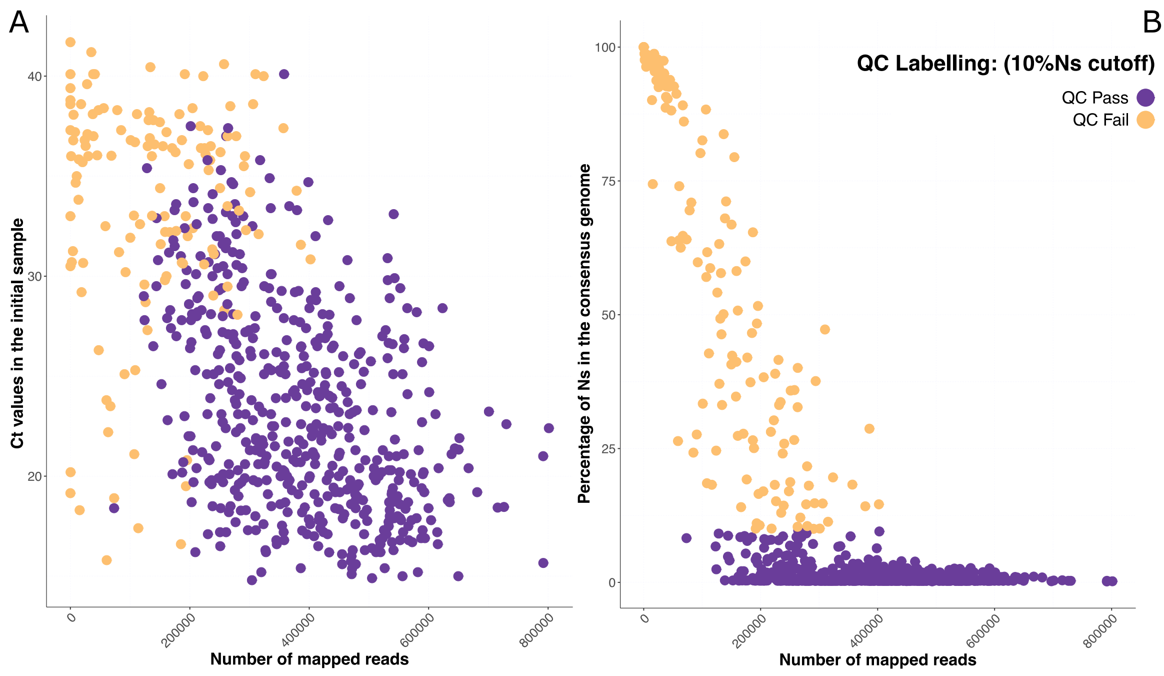

Figure S6. COVGAP evaluation of sequencing quality parameters.

Of the original 746 samples, 689 successfully sequenced. Number of mapped reads across all SARS-CoV-2 positive samples successfully sequenced from 26th of February till 23th of March: (n=689), of which 533 passed the quality filter, and 156 failed. 468 of the samples passing the quality filters were matching the cohort eligibility criteria and therefore were further described in the present study. **A.** Number of mapped reads against Ct values from diagnostic tests; **B.** Number of mapped reads against percentage of Ns in the consensus.
