# Supplementary material for: SARS-CoV-2 outbreak in a tri-national urban area is dominated by a B.1 lineage variant linked to a mass gathering event
Source: PLoS Pathog. 2021 Mar 19;17(3):e1009374. doi: 10.1371/journal.ppat.1009374 (PMC8011817; doi:10.1371/journal.ppat.1009374)
Supplement: S1 Table — Each observation consists of the genome position multiplied by the number of samples in which it appears. (PDF) [file ppat.1009374.s007.pdf]

Table S1. Counts and description of the in silico mutated genome community used for COVGAP validation. Each observation consists of the genome position multiplied by the number of samples in which it appears.

Position	Type	Bp length	Mock input	Covgap output	Genome length	Mock positive	Covgap positive	True positive (tp)	False positive (fp)	Mock negative	Covgap negative	True negative (tn)	False negtive (fn)
**241**	snp	1	1	1	29903	1	1	1	0	29902	29902	29902	0
**2247**	del	3	4	4	29903	12	12	12	0	119600	119600	119600	0
**3022**	snp	1	1	1	29903	1	1	1	0	29902	29902	29902	0
**4710**	snp	1	4	4	29903	4	4	4	0	119608	119608	119608	0
**5845**	del	1	3	0	29903	3	0	0	0	89706	89709	89706	3
**9082**	snp	1	2	2	29903	2	2	2	0	59804	59804	59804	0
**9504**	snp	1	3	3	29903	3	3	3	0	89706	89706	89706	0
**11661**	del	3	4	4	29903	12	12	12	0	119600	119600	119600	0
**12113**	del	1	1	1	29903	1	1	1	0	29902	29902	29902	0
**14392**	snp	1	1	1	29903	1	1	1	0	29902	29902	29902	0
**14408**	snp	1	1	1	29903	1	1	1	0	29902	29902	29902	0
**14850**	snp	1	4	4	29903	4	4	4	0	119608	119608	119608	0
**14872**	snp	1	1	1	29903	1	1	1	0	29902	29902	29902	0
**15095**	snp	1	1	1	29903	1	1	1	0	29902	29902	29902	0
**15265**	snp	1	1	1	29903	1	1	1	0	29902	29902	29902	0
**15272**	snp	1	2	2	29903	2	2	2	0	59804	59804	59804	0
**15283**	snp	1	1	1	29903	1	1	1	0	29902	29902	29902	0
**15907**	snp	1	2	2	29903	2	2	2	0	59804	59804	59804	0
**16145**	ins	3	1	0	29903	3	0	0	0	29900	29903	29900	3
**16281**	del	1	5	0	29903	5	0	0	0	149510	149515	149510	5
**16600**	snp	1	1	1	29903	1	1	1	0	29902	29902	29902	0
**17258**	snp	1	4	4	29903	4	4	4	0	119608	119608	119608	0
**18315**	snp	1	5	5	29903	5	5	5	0	149510	149510	149510	0
**18837**	ins	3	4	4	29903	12	12	12	0	119600	119600	119600	0
**19653**	snp	1	4	4	29903	4	4	4	0	119608	119608	119608	0
**21795**	snp	1	5	5	29903	5	5	5	0	149510	149510	149510	0
**21812**	snp	1	4	4	29903	4	4	4	0	119608	119608	119608	0
**23388**	snp	1	1	1	29903	1	1	1	0	29902	29902	29902	0
**23682**	snp	1	4	4	29903	4	4	4	0	119608	119608	119608	0
**26852**	snp	1	1	1	29903	1	1	1	0	29902	29902	29902	0
**26907**	snp	1	1	1	29903	1	1	1	0	29902	29902	29902	0
**27405**	del	81	1	1	29903	81	81	81	0	29822	29822	29822	0
**27528**	snp	1	1	1	29903	1	1	1	0	29902	29902	29902	0
**28341**	snp	1	2	2	29903	2	2	2	0	59804	59804	59804	0
**28881**	snp	1	1	1	29903	1	1	1	0	29902	29902	29902	0

Position	Type	Bp length	Mock input	Covgap output	Genome length	Mock positive	Covgap positive	True positive (tp)	False positive (fp)	Mock negative	Covgap negative	True negative (tn)	False negtive (fn)
**28882**	snp	1	1	1	29903	1	1	1	0	29902	29902	29902	0
**28883**	snp	1	1	1	29903	1	1	1	0	29902	29902	29902	0
**29165**	snp	1	1	1	29903	1	1	1	0	29902	29902	29902	0
