# Supplementary material for: SARS-CoV-2 outbreak in a tri-national urban area is dominated by a B.1 lineage variant linked to a mass gathering event
Source: PLoS Pathog. 2021 Mar 19;17(3):e1009374. doi: 10.1371/journal.ppat.1009374 (PMC8011817; doi:10.1371/journal.ppat.1009374)
Supplement: S2 Table — (PDF) [file ppat.1009374.s008.pdf]

Table S2. Diversity indices for SARS-CoV-2 lineages in Switzerland and neighbouring countries for the time period of first detected case until March 23rd, 2020.

Country	Coefficient of co-variation	Shannon Entropy H’	Shannon Diversity	Simpson Concentration Index D’	Simpson Diversity
**Austria**	1.654	1.704	5.496	0.265	3.772
**France**	1.987	0.656	1.927	0.701	1.426
**Germany**	1.305	1.654	5.226	0.246	4.066
**Italy**	0.749	1.067	2.908	0.396	2.523
**Switzerland**	2.783	0.866	2.377	0.622	1.609
