# Supplementary material for: SARS-CoV-2 outbreak in a tri-national urban area is dominated by a B.1 lineage variant linked to a mass gathering event
Source: PLoS Pathog. 2021 Mar 19;17(3):e1009374. doi: 10.1371/journal.ppat.1009374 (PMC8011817; doi:10.1371/journal.ppat.1009374)
Supplement: S3 Table — (PDF) [file ppat.1009374.s009.pdf]

Table S3. List of countries that recorded genomes with mutation C15324T in B.1 background and number of total genomes sequenced until March 23rd 2020.

Country	Number genomes with C15324T	Total genomes sequenced until march 23rd	% genomes with mutation	% of population sequenced	Population
**ARGENTINA**	1	4	25.00	0.00001	45,195,774
**AUSTRALIA**	14	1092	1.28	0.00428	25,499,884
**AUSTRIA**	3	244	1.23	0.00271	9,006,398
**BELGIUM**	40	268	14.93	0.00231	11,589,623
**BENIN**	1	6	16.67	0.00005	12,123,200
**BOSNIA AND HERZEGOVINA**	2	12	16.67	0.00037	3,280,819
**BRAZIL**	1	226	0.44	0.00011	212,559,417
**CANADA**	7	405	1.73	0.00107	37,742,154
**CHILE**	2	120	1.67	0.00063	19,116,201
**COSTA RICA**	1	40	2.50	0.00079	5,094,118
**DEMOCRATIC REPUBLIC OF THE CONGO**	11	35	31.43	0.00004	89,561,403
**ENGLAND**	4	5643 (UK)	0.01	0.00831	67,886,011
**FRANCE**	69	369	18.70	0.00057	65,273,511
**GERMANY**	2	147	1.36	0.00018	83,783,942
**HUNGARY**	2	18	11.11	0.00019	9,660,351
**ICELAND**	5	522	0.96	0.15297	341,243
**INDIA**	1	119	0.84	0.00001	1,380,004,385
**ISRAEL**	1	72	1.39	0.00083	8,655,535
**JAPAN**	3	343	0.87	0.00027	126,476,461
**LUXEMBOURG**	24	116	20.69	0.01853	625,978
**MOROCCO**	3	13	23.08	0.00004	36,910,560
**NETHERLANDS**	2	617	0.32	0.00360	17,134,872
**OMAN**	1	21	4.76	0.00041	5,106,626
**PORTUGAL**	8	570	1.40	0.00559	10,196,709
**RUSSIA**	1	59	1.69	0.00004	145,934,462
**SCOTLAND**	4	5643 (UK)	0.01	0.00831	67,886,011
**SENEGAL**	3	24	12.50	0.00014	16,743,927
**SOUTH KOREA**	1	196	0.51	0.00038	51,269,185
**SWITZERLAND**	57 (386)*	213 (675)*	26.8 (57.2)*	0.00780	8,654,622
**TAIWAN**	3	95	3.16	0.00040	23,816,775
**USA**	1	4150	0.02	0.00125	331,002,651
**VIETNAM**	1	59	1.69	0.00006	97,338,579

**** Number in brackets summarize counts of genomes from GISAID plus genomes from this study***
