# Supplementary material for: SARS-CoV-2 outbreak in a tri-national urban area is dominated by a B.1 lineage variant linked to a mass gathering event
Source: PLoS Pathog. 2021 Mar 19;17(3):e1009374. doi: 10.1371/journal.ppat.1009374 (PMC8011817; doi:10.1371/journal.ppat.1009374)
Supplement: S4 Table — (PDF) [file ppat.1009374.s010.pdf]

Table S4. GISAID identifiers and dates of sampling for all sequences that belong to emerging clade 20A/15324T with a collection date until March 23rd, 2020 (N = 279).

GISAID identifier of genomes that belong to 20A/15324T	collection date	Country	Canton if in Switzerland
hCoV-19/Germany/FrankfurtFFM7/2020|EPI_ISL_452223|2020-03-02	2020-03-02	Germany	
hCoV-19/France/HF1870/2020|EPI_ISL_414629|2020-03-03	2020-03-03	France	
hCoV-19/France/Valence_532/2020|EPI_ISL_416749|2020-03-04	2020-03-04	France	
hCoV-19/Switzerland/Bern2536/2020|EPI_ISL_415704|2020-03-04	2020-03-04	Switzerland	Basel-Landschaft
hCoV-19/France/Lyon_06056/2020|EPI_ISL_417334|2020-03-04	2020-03-04	France	
hCoV-19/France/CVL2000/2020|EPI_ISL_418222|2020-03-04	2020-03-04	France	
hCoV-19/France/Clermont-Ferrand_650/2020|EPI_ISL_416752|2020-03-04	2020-03-04	France	
hCoV-19/France/Lyon_487/2020|EPI_ISL_416747|2020-03-04	2020-03-04	France	
hCoV-19/France/Lyon_06042/2020|EPI_ISL_417333|2020-03-04	2020-03-04	France	
hCoV-19/Austria/CeMM0449/2020|EPI_ISL_475866|2020-03-05	2020-03-05	Austria	
hCoV-19/France/BFC2147/2020|EPI_ISL_415652|2020-03-05	2020-03-05	France	
hCoV-19/France/BFC2094/2020|EPI_ISL_415651|2020-03-05	2020-03-05	France	
hCoV-19/France/Clermont-Ferrand_651/2020|EPI_ISL_416751|2020-03-05	2020-03-05	France	
hCoV-19/Switzerland/100010/2020|EPI_ISL_466935|2020-03-05	2020-03-05	Switzerland	Basel-Landschaft
hCoV-19/Switzerland/GE4135/2020|EPI_ISL_415705|2020-03-06	2020-03-06	Switzerland	Geneva
hCoV-19/Belgium/NKR-030645/2020|EPI_ISL_418795|2020-03-06	2020-03-06	Belgium	
hCoV-19/France/Lyon_06487/2020|EPI_ISL_416754|2020-03-06	2020-03-06	France	
hCoV-19/France/Lyon_06531/2020|EPI_ISL_416756|2020-03-06	2020-03-06	France	
hCoV-19/Switzerland/100006/2020|EPI_ISL_466931|2020-03-06	2020-03-06	Switzerland	Basel-City
hCoV-19/France/Lyon_683/2020|EPI_ISL_416750|2020-03-06	2020-03-06	France	
hCoV-19/Switzerland/100005/2020|EPI_ISL_466930|2020-03-06	2020-03-06	Switzerland	Basel-Landschaft
hCoV-19/Belgium/ULG-10122/2020|EPI_ISL_427370|2020-03-07	2020-03-07	Belgium	
hCoV-19/France/Lyon_06625/2020|EPI_ISL_417337|2020-03-07	2020-03-07	France	
hCoV-19/France/Bourg-en-Bresse_06678/2020|EPI_ISL_416757|2020-03-07	2020-03-07	France	
hCoV-19/France/Lyon_06820/2020|EPI_ISL_417339|2020-03-08	2020-03-08	France	
hCoV-19/Austria/CeMM0390/2020|EPI_ISL_475830|2020-03-08	2020-03-08	Austria	
hCoV-19/Switzerland/GE6679/2020|EPI_ISL_415707|2020-03-08	2020-03-08	Switzerland	Geneva
hCoV-19/France/HF2196/2020|EPI_ISL_416493|2020-03-08	2020-03-08	France	
hCoV-19/Luxembourg/LNS2614631/2020|EPI_ISL_419578|2020-03-08	2020-03-08	Luxembourg	
hCoV-19/Switzerland/100018/2020|EPI_ISL_466942|2020-03-08	2020-03-08	Switzerland	Basel-City
hCoV-19/Switzerland/100017/2020|EPI_ISL_466941|2020-03-08	2020-03-08	Switzerland	Basel-City
hCoV-19/France/ARA06923/2020|EPI_ISL_508937|2020-03-09	2020-03-09	France	
hCoV-19/Israel/CVL-n3120/2020|EPI_ISL_447251|2020-03-09	2020-03-09	Israel	
hCoV-19/Belgium/FAE-030948/2020|EPI_ISL_420445|2020-03-09	2020-03-09	Belgium	
hCoV-19/Belgium/RDC-030961/2020|EPI_ISL_420452|2020-03-09	2020-03-09	Belgium	
hCoV-19/Japan/PG-0233/2020|EPI_ISL_479928|2020-03-09	2020-03-09	Japan	

GISAID identifier of genomes that belong to 20A/15324T	collection date	Country	Canton if in Switzerland
hCoV-19/Japan/PG-0234/2020|EPI_ISL_479929|2020-03-09	2020-03-09	Japan	
hCoV-19/Japan/PG-0235/2020|EPI_ISL_479930|2020-03-09	2020-03-09	Japan	
hCoV-19/Canada/NB_1/2020|EPI_ISL_429806|2020-03-09	2020-03-09	Canada	
hCoV-19/Switzerland/100021/2020|EPI_ISL_466945|2020-03-09	2020-03-09	Switzerland	Basel-Landschaft
hCoV-19/France/ARA07314/2020|EPI_ISL_508942|2020-03-10	2020-03-10	France	
hCoV-19/Switzerland/100023/2020|EPI_ISL_466947|2020-03-10	2020-03-10	Switzerland	Bern
hCoV-19/Switzerland/100029/2020|EPI_ISL_466953|2020-03-10	2020-03-10	Switzerland	Basel-City
hCoV-19/Switzerland/100025/2020|EPI_ISL_466949|2020-03-10	2020-03-10	Switzerland	Basel-City
hCoV-19/Switzerland/100027/2020|EPI_ISL_466951|2020-03-10	2020-03-10	Switzerland	Basel-City
hCoV-19/France/Pollionay_1733/2020|EPI_ISL_416745|2020-03-10	2020-03-10	France	
hCoV-19/Switzerland/100028/2020|EPI_ISL_466952|2020-03-10	2020-03-10	Switzerland	Basel-City
hCoV-19/Switzerland/100032/2020|EPI_ISL_466956|2020-03-10	2020-03-10	Switzerland	Zurich
hCoV-19/Switzerland/100024/2020|EPI_ISL_466948|2020-03-10	2020-03-10	Switzerland	Valais
hCoV-19/Switzerland/100022/2020|EPI_ISL_466946|2020-03-10	2020-03-10	Switzerland	Basel-City
hCoV-19/Luxembourg/LNS4691488/2020|EPI_ISL_419587|2020-03-11	2020-03-11	Luxembourg	
hCoV-19/Australia/VIC38/2020|EPI_ISL_419756|2020-03-11	2020-03-11	Australia	
hCoV-19/Switzerland/100036/2020|EPI_ISL_466960|2020-03-11	2020-03-11	Switzerland	Basel-City
hCoV-19/France/IDF2278/2020|EPI_ISL_416499|2020-03-11	2020-03-11	France	
hCoV-19/England/201160276/2020|EPI_ISL_464795|2020-03-11	2020-03-11	England	
hCoV-19/Switzerland/100034/2020|EPI_ISL_466958|2020-03-11	2020-03-11	Switzerland	Basel-City
hCoV-19/Switzerland/100041/2020|EPI_ISL_466964|2020-03-11	2020-03-11	Switzerland	Zurich
hCoV-19/Germany/BW-RKI-N-0001/2020|EPI_ISL_481253|2020-03-12	2020-03-12	Germany	
hCoV-19/Switzerland/100076/2020|EPI_ISL_451669|2020-03-12	2020-03-12	Switzerland	Vaud
hCoV-19/Canada/NB_6/2020|EPI_ISL_418811|2020-03-12	2020-03-12	Canada	
hCoV-19/Luxembourg/LNS1234709/2020|EPI_ISL_419570|2020-03-12	2020-03-12	Luxembourg	
hCoV-19/Luxembourg/LNS2907333/2020|EPI_ISL_419580|2020-03-12	2020-03-12	Luxembourg	
hCoV-19/France/GE2720/2020|EPI_ISL_420039|2020-03-12	2020-03-12	France	
hCoV-19/Canada/NB_21/2020|EPI_ISL_429811|2020-03-12	2020-03-12	Canada	
hCoV-19/Luxembourg/LNS0156959/2020|EPI_ISL_419563|2020-03-12	2020-03-12	Luxembourg	
hCoV-19/France/GE2722/2020|EPI_ISL_420040|2020-03-12	2020-03-12	France	
hCoV-19/Austria/CeMM0109/2020|EPI_ISL_437968|2020-03-12	2020-03-12	Austria	
hCoV-19/Iceland/133/2020|EPI_ISL_417788|2020-03-12	2020-03-12	Iceland	
hCoV-19/Switzerland/100054/2020|EPI_ISL_466976|2020-03-12	2020-03-12	Switzerland	Bern
hCoV-19/Switzerland/100058/2020|EPI_ISL_466980|2020-03-12	2020-03-12	Switzerland	Vaud
hCoV-19/Switzerland/100055/2020|EPI_ISL_466977|2020-03-12	2020-03-12	Switzerland	Bern
hCoV-19/Switzerland/100082/2020|EPI_ISL_451674|2020-03-13	2020-03-13	Switzerland	Zurich
hCoV-19/France/IDF2420/2020|EPI_ISL_418230|2020-03-13	2020-03-13	France	
hCoV-19/Australia/NSW2147/2020|EPI_ISL_500628|2020-03-13	2020-03-13	Australia	
hCoV-19/Switzerland/100081/2020|EPI_ISL_451673|2020-03-13	2020-03-13	Switzerland	Basel-Landschaft
hCoV-19/Switzerland/100083/2020|EPI_ISL_451675|2020-03-13	2020-03-13	Switzerland	Basel-City
hCoV-19/Switzerland/100086/2020|EPI_ISL_451678|2020-03-13	2020-03-13	Switzerland	Basel-Landschaft

GISAID identifier of genomes that belong to 20A/15324T	collection date	Country	Canton if in Switzerland
hCoV-19/Switzerland/100091/2020|EPI_ISL_451683|2020-03-13	2020-03-13	Switzerland	Basel-Landschaft
hCoV-19/Switzerland/100093/2020|EPI_ISL_451685|2020-03-13	2020-03-13	Switzerland	Zurich
hCoV-19/Senegal/139/2020|EPI_ISL_418217|2020-03-13	2020-03-13	Senegal	
hCoV-19/France/IDF2534/2020|EPI_ISL_418234|2020-03-14	2020-03-14	France	
hCoV-19/Belgium/ULG-7729/2020|EPI_ISL_418630|2020-03-14	2020-03-14	Belgium	
hCoV-19/Oman/RESP-20-2282/2020|EPI_ISL_457974|2020-03-14	2020-03-14	Oman	
hCoV-19/Luxembourg/LNS8188502/2020|EPI_ISL_419598|2020-03-14	2020-03-14	Luxembourg	
hCoV-19/France/IDF2536/2020|EPI_ISL_421504|2020-03-14	2020-03-14	France	
hCoV-19/Switzerland/1000503309/2020|EPI_ISL_509222|2020-03-14	2020-03-14	Switzerland	Zurich
hCoV-19/Luxembourg/LNS0756270/2020|EPI_ISL_419568|2020-03-14	2020-03-14	Luxembourg	
hCoV-19/Switzerland/1000482984/2020|EPI_ISL_508866|2020-03-14	2020-03-14	Switzerland	Zurich
hCoV-19/Canada/NB_35/2020|EPI_ISL_429816|2020-03-14	2020-03-14	Canada	
hCoV-19/Luxembourg/LNS2886370/2020|EPI_ISL_419579|2020-03-14	2020-03-14	Luxembourg	
hCoV-19/Belgium/ULG-7134/2020|EPI_ISL_418628|2020-03-14	2020-03-14	Belgium	
hCoV-19/Switzerland/100109/2020|EPI_ISL_451693|2020-03-14	2020-03-14	Switzerland	Basel-Landschaft
hCoV-19/France/ARA094100/2020|EPI_ISL_418412|2020-03-15	2020-03-15	France	
hCoV-19/Chile/Valparaiso_1/2020|EPI_ISL_445272|2020-03-15	2020-03-15	Chile	
hCoV-19/Benin/197/2020|EPI_ISL_476822|2020-03-15	2020-03-15	Benin	
hCoV-19/France/HF2496/2020|EPI_ISL_418231|2020-03-15	2020-03-15	France	
hCoV-19/Switzerland/1000482982/2020|EPI_ISL_508864|2020-03-15	2020-03-15	Switzerland	Zurich
hCoV-19/Switzerland/1000482983/2020|EPI_ISL_508865|2020-03-15	2020-03-15	Switzerland	Zurich
hCoV-19/Switzerland/1000483258/2020|EPI_ISL_508868|2020-03-15	2020-03-15	Switzerland	Zurich
hCoV-19/France/ARA09178/2020|EPI_ISL_508877|2020-03-15	2020-03-15	France	
hCoV-19/South Korea/KCDC2125/2020|EPI_ISL_498026|2020-03-15	2020-03-15	South Korea	
hCoV-19/Canada/NB_56/2020|EPI_ISL_482473|2020-03-15	2020-03-15	Canada	
hCoV-19/Iceland/192/2020|EPI_ISL_417819|2020-03-15	2020-03-15	Iceland	
hCoV-19/Iceland/155/2020|EPI_ISL_417737|2020-03-15	2020-03-15	Iceland	
hCoV-19/France/ARA09434/2020|EPI_ISL_418414|2020-03-15	2020-03-15	France	
hCoV-19/Australia/SAP220/2020|EPI_ISL_467995|2020-03-15	2020-03-15	Australia	
hCoV-19/Taiwan/NTU12/2020|EPI_ISL_422414|2020-03-16	2020-03-16	Taiwan	
hCoV-19/Luxembourg/LNS6603085/2020|EPI_ISL_419593|2020-03-16	2020-03-16	Luxembourg	
hCoV-19/Switzerland/100133/2020|EPI_ISL_451717|2020-03-16	2020-03-16	Switzerland	Aargau
hCoV-19/Switzerland/100111/2020|EPI_ISL_451695|2020-03-16	2020-03-16	Switzerland	Basel-City
hCoV-19/Switzerland/1000484569/2020|EPI_ISL_508869|2020-03-16	2020-03-16	Switzerland	Zurich
hCoV-19/Switzerland/100130/2020|EPI_ISL_451714|2020-03-16	2020-03-16	Switzerland	Vaud
hCoV-19/Switzerland/100114/2020|EPI_ISL_451698|2020-03-16	2020-03-16	Switzerland	Basel-Landschaft
hCoV-19/Switzerland/100137/2020|EPI_ISL_451721|2020-03-16	2020-03-16	Switzerland	Basel-Landschaft
hCoV-19/France/ARA10242/2020|EPI_ISL_508881|2020-03-16	2020-03-16	France	
hCoV-19/France/ARA10175/2020|EPI_ISL_508880|2020-03-16	2020-03-16	France	
hCoV-19/France/ARA10160/2020|EPI_ISL_508879|2020-03-16	2020-03-16	France	
hCoV-19/France/IDF2684/2020|EPI_ISL_418240|2020-03-16	2020-03-16	France	

GISAID identifier of genomes that belong to 20A/15324T	collection date	Country	Canton if in Switzerland
hCoV-19/France/ARA10163/2020|EPI_ISL_418418|2020-03-16	2020-03-16	France	
hCoV-19/Switzerland/100113/2020|EPI_ISL_451697|2020-03-16	2020-03-16	Switzerland	Bern
hCoV-19/Switzerland/100117/2020|EPI_ISL_451701|2020-03-16	2020-03-16	Switzerland	Bern
hCoV-19/Switzerland/100118/2020|EPI_ISL_451702|2020-03-16	2020-03-16	Switzerland	Bern
hCoV-19/Switzerland/100124/2020|EPI_ISL_451708|2020-03-16	2020-03-16	Switzerland	Jura
hCoV-19/Switzerland/100125/2020|EPI_ISL_451709|2020-03-16	2020-03-16	Switzerland	Aargau
hCoV-19/Switzerland/100129/2020|EPI_ISL_451713|2020-03-16	2020-03-16	Switzerland	Basel-City
hCoV-19/Switzerland/100139/2020|EPI_ISL_451723|2020-03-16	2020-03-16	Switzerland	Grisons
hCoV-19/Switzerland/100144/2020|EPI_ISL_451728|2020-03-16	2020-03-16	Switzerland	Basel-City
hCoV-19/France/ARA10165/2020|EPI_ISL_418419|2020-03-16	2020-03-16	France	
hCoV-19/Switzerland/100120/2020|EPI_ISL_451704|2020-03-16	2020-03-16	Switzerland	Jura
hCoV-19/Switzerland/100112/2020|EPI_ISL_451696|2020-03-16	2020-03-16	Switzerland	Basel-City
hCoV-19/Switzerland/100136/2020|EPI_ISL_451720|2020-03-16	2020-03-16	Switzerland	Basel-Landschaft
hCoV-19/Portugal/PT0039/2020|EPI_ISL_418024|2020-03-17	2020-03-17	Portugal	
hCoV-19/Belgium/GE-0317105/2020|EPI_ISL_420361|2020-03-17	2020-03-17	Belgium	
hCoV-19/Belgium/ULG-10099/2020|EPI_ISL_427350|2020-03-17	2020-03-17	Belgium	
hCoV-19/Luxembourg/LNS2299410/2020|EPI_ISL_421745|2020-03-17	2020-03-17	Luxembourg	
hCoV-19/Luxembourg/LNS3035957/2020|EPI_ISL_421750|2020-03-17	2020-03-17	Luxembourg	
hCoV-19/France/BFC2709/2020|EPI_ISL_420038|2020-03-17	2020-03-17	France	
hCoV-19/France/IDF2848/2020|EPI_ISL_420046|2020-03-17	2020-03-17	France	
hCoV-19/Belgium/HN-0317324/2020|EPI_ISL_462178|2020-03-17	2020-03-17	Belgium	
hCoV-19/Belgium/HMM-0317327/2020|EPI_ISL_462181|2020-03-17	2020-03-17	Belgium	
hCoV-19/Luxembourg/LNS0819394/2020|EPI_ISL_421746|2020-03-17	2020-03-17	Luxembourg	
hCoV-19/Luxembourg/LNS4569788/2020|EPI_ISL_421752|2020-03-17	2020-03-17	Luxembourg	
hCoV-19/Luxembourg/LNS7991123/2020|EPI_ISL_421753|2020-03-17	2020-03-17	Luxembourg	
hCoV-19/Luxembourg/LNS3404815/2020|EPI_ISL_421738|2020-03-17	2020-03-17	Luxembourg	
hCoV-19/Switzerland/GE2164/2020|EPI_ISL_429208|2020-03-17	2020-03-17	Switzerland	Geneva
hCoV-19/Belgium/UGent-97/2020|EPI_ISL_468745|2020-03-17	2020-03-17	Belgium	
hCoV-19/Belgium/ULG-7500/2020|EPI_ISL_418632|2020-03-17	2020-03-17	Belgium	
hCoV-19/Belgium/ULG-8532/2020|EPI_ISL_418635|2020-03-17	2020-03-17	Belgium	
hCoV-19/Chile/Santiago_32/2020|EPI_ISL_445311|2020-03-17	2020-03-17	Chile	
hCoV-19/Costa Rica/INC-0048/2020|EPI_ISL_512654|2020-03-17	2020-03-17	Costa Rica	
hCoV-19/France/ARA10188/2020|EPI_ISL_418423|2020-03-17	2020-03-17	France	
hCoV-19/DRC/KN-0058/2020|EPI_ISL_417438|2020-03-17	2020-03-17	DRC	
hCoV-19/Portugal/PT0702/2020|EPI_ISL_510961|2020-03-17	2020-03-17	Portugal	
hCoV-19/Portugal/PT0703/2020|EPI_ISL_510962|2020-03-17	2020-03-17	Portugal	
hCoV-19/France/GE3067/2020|EPI_ISL_420055|2020-03-17	2020-03-17	France	
hCoV-19/Morocco/6890/2020|EPI_ISL_459968|2020-03-17	2020-03-17	Morocco	
hCoV-19/Portugal/PT0700/2020|EPI_ISL_510959|2020-03-17	2020-03-17	Portugal	
hCoV-19/France/ARA10257/2020|EPI_ISL_418427|2020-03-17	2020-03-17	France	
hCoV-19/DRC/KN-0054/2020|EPI_ISL_417437|2020-03-17	2020-03-17	DRC	
hCoV-19/Senegal/315/2020|EPI_ISL_420070|2020-03-17	2020-03-17	Senegal	

GISAID identifier of genomes that belong to 20A/15324T	collection date	Country	Canton if in Switzerland
hCoV-19/Portugal/PT0701/2020|EPI_ISL_510960|2020-03-17	2020-03-17	Portugal	
hCoV-19/France/ARA10189/2020|EPI_ISL_418424|2020-03-17	2020-03-17	France	
hCoV-19/Belgium/UGent-95/2020|EPI_ISL_468743|2020-03-17	2020-03-17	Belgium	
hCoV-19/France/ARA10184/2020|EPI_ISL_418422|2020-03-17	2020-03-17	France	
hCoV-19/England/CAMB-7507F/2020|EPI_ISL_440405|2020-03-17	2020-03-17	England	
hCoV-19/DRC/KN-0070/2020|EPI_ISL_417441|2020-03-17	2020-03-17	DRC	
hCoV-19/Luxembourg/LNS0158952/2020|EPI_ISL_417534|2020-03-18	2020-03-18	Luxembourg	
hCoV-19/Belgium/Vaud-0318102/2020|EPI_ISL_420358|2020-03-18	2020-03-18	Belgium	
hCoV-19/Belgium/UM-0318103/2020|EPI_ISL_420359|2020-03-18	2020-03-18	Belgium	
hCoV-19/Belgium/VL-0318104/2020|EPI_ISL_420360|2020-03-18	2020-03-18	Belgium	
hCoV-19/Luxembourg/LNS9371718/2020|EPI_ISL_417526|2020-03-18	2020-03-18	Luxembourg	
hCoV-19/Luxembourg/LNS2848109/2020|EPI_ISL_417527|2020-03-18	2020-03-18	Luxembourg	
hCoV-19/France/ARA10580/2020|EPI_ISL_508999|2020-03-18	2020-03-18	France	
hCoV-19/Switzerland/GE3144/2020|EPI_ISL_429196|2020-03-18	2020-03-18	Switzerland	Geneva
hCoV-19/France/ARA10821/2020|EPI_ISL_509000|2020-03-18	2020-03-18	France	
hCoV-19/Australia/VIC170/2020|EPI_ISL_419867|2020-03-18	2020-03-18	Australia	
hCoV-19/DRC/82/2020|EPI_ISL_417946|2020-03-18	2020-03-18	DRC	
hCoV-19/Belgium/20A30/2020|EPI_ISL_451935|2020-03-18	2020-03-18	Belgium	
hCoV-19/Belgium/UGent-110/2020|EPI_ISL_475070|2020-03-18	2020-03-18	Belgium	
hCoV-19/Portugal/PT0044/2020|EPI_ISL_421447|2020-03-18	2020-03-18	Portugal	
hCoV-19/Portugal/PT0793/2020|EPI_ISL_511052|2020-03-18	2020-03-18	Portugal	
hCoV-19/Taiwan/128/2020|EPI_ISL_420083|2020-03-18	2020-03-18	Taiwan	
hCoV-19/France/HF2797/2020|EPI_ISL_420044|2020-03-18	2020-03-18	France	
hCoV-19/France/ARA10823/2020|EPI_ISL_508882|2020-03-18	2020-03-18	France	
hCoV-19/DRC/80/2020|EPI_ISL_417942|2020-03-18	2020-03-18	DRC	
hCoV-19/DRC/KN-0072/2020|EPI_ISL_417442|2020-03-18	2020-03-18	DRC	
hCoV-19/Belgium/UGent-102/2020|EPI_ISL_475062|2020-03-18	2020-03-18	Belgium	
hCoV-19/DRC/73/2020|EPI_ISL_417941|2020-03-18	2020-03-18	DRC	
hCoV-19/Luxembourg/LNS9984061/2020|EPI_ISL_429724|2020-03-19	2020-03-19	Luxembourg	
hCoV-19/Belgium/OT-0319109/2020|EPI_ISL_420365|2020-03-19	2020-03-19	Belgium	
hCoV-19/Hungary/US-1791lc/2020|EPI_ISL_477615|2020-03-19	2020-03-19	Hungary	
hCoV-19/DRC/94/2020|EPI_ISL_417947|2020-03-19	2020-03-19	DRC	
hCoV-19/Switzerland/1000486784/2020|EPI_ISL_508870|2020-03-19	2020-03-19	Switzerland	Zurich
hCoV-19/Belgium/ULG-8595/2020|EPI_ISL_418634|2020-03-19	2020-03-19	Belgium	
hCoV-19/Russia/Sverdlovsk-71903/2020|EPI_ISL_428890|2020-03-19	2020-03-19	Russia	
hCoV-19/Bosnia and Herzegovina/ChVir7343/2020|EPI_ISL_462451|2020-03-19	2020-03-19	Bosnia and Herzegovina	
hCoV-19/Portugal/PT0508/2020|EPI_ISL_454232|2020-03-19	2020-03-19	Portugal	
hCoV-19/Belgium/CA-0319111/2020|EPI_ISL_420368|2020-03-19	2020-03-19	Belgium	
hCoV-19/Belgium/ULG-9619/2020|EPI_ISL_418649|2020-03-19	2020-03-19	Belgium	
hCoV-19/Scotland/CVR219/2020|EPI_ISL_425746|2020-03-19	2020-03-19	Scotland	
hCoV-19/Iceland/398/2020|EPI_ISL_424422|2020-03-19	2020-03-19	Iceland	
hCoV-19/Australia/SAP034/2020|EPI_ISL_451079|2020-03-19	2020-03-19	Australia	

GISAID identifier of genomes that belong to 20A/15324T	collection date	Country	Canton if in Switzerland
hCoV-19/Morocco/6894/2020|EPI_ISL_459972|2020-03-20	2020-03-20	Morocco	
hCoV-19/Morocco/6896/2020|EPI_ISL_459974|2020-03-20	2020-03-20	Morocco	
hCoV-19/France/GE3372/2020|EPI_ISL_428348|2020-03-20	2020-03-20	France	
hCoV-19/France/GE4420/2020|EPI_ISL_443310|2020-03-20	2020-03-20	France	
hCoV-19/England/LIVE-A5498/2020|EPI_ISL_500119|2020-03-20	2020-03-20	England	
hCoV-19/Belgium/Rega-0320258/2020|EPI_ISL_458228|2020-03-20	2020-03-20	Belgium	
hCoV-19/Iceland/435/2020|EPI_ISL_424459|2020-03-20	2020-03-20	Iceland	
hCoV-19/Belgium/ULG-9558/2020|EPI_ISL_418645|2020-03-21	2020-03-21	Belgium	
hCoV-19/DRC/214/2020|EPI_ISL_420030|2020-03-21	2020-03-21	DRC	
hCoV-19/France/IDF3165/2020|EPI_ISL_420059|2020-03-21	2020-03-21	France	
hCoV-19/Senegal/659/2020|EPI_ISL_480554|2020-03-21	2020-03-21	Senegal	
hCoV-19/France/IDF3236/2020|EPI_ISL_421506|2020-03-21	2020-03-21	France	
hCoV-19/France/ARA12155/2020|EPI_ISL_508949|2020-03-21	2020-03-21	France	
hCoV-19/India/NIV-4130/2020|EPI_ISL_454529|2020-03-21	2020-03-21	India	
hCoV-19/Bosnia and Herzegovina/ChVir7352/2020|EPI_ISL_462458|2020-03-21	2020-03-21	Bosnia and Herzegovina	
hCoV-19/Netherlands/NoordBrabant_102/2020|EPI_ISL_422861|2020-03-21	2020-03-21	Netherlands	
hCoV-19/France/ARA11943/2020|EPI_ISL_419170|2020-03-21	2020-03-21	France	
hCoV-19/Hungary/US-2587w/2020|EPI_ISL_477618|2020-03-22	2020-03-22	Hungary	
hCoV-19/Belgium/ULG-10094/2020|EPI_ISL_424650|2020-03-22	2020-03-22	Belgium	
hCoV-19/Australia/VIC137/2020|EPI_ISL_419830|2020-03-22	2020-03-22	Australia	
hCoV-19/Canada/NL_95/2020|EPI_ISL_469234|2020-03-22	2020-03-22	Canada	
hCoV-19/Canada/NL_97/2020|EPI_ISL_469236|2020-03-22	2020-03-22	Canada	
hCoV-19/Belgium/ULG-9716/2020|EPI_ISL_418657|2020-03-22	2020-03-22	Belgium	
hCoV-19/USA/WI-UW-37/2020|EPI_ISL_421295|2020-03-22	2020-03-22	USA	
hCoV-19/Belgium/ULG-9618/2020|EPI_ISL_418648|2020-03-22	2020-03-22	Belgium	
hCoV-19/Belgium/ULG-9715/2020|EPI_ISL_418656|2020-03-22	2020-03-22	Belgium	
hCoV-19/Argentina/C3013/2020|EPI_ISL_420598|2020-03-22	2020-03-22	Argentina	
hCoV-19/France/ARA12264/2020|EPI_ISL_419185|2020-03-22	2020-03-22	France	
hCoV-19/France/ARA12238/2020|EPI_ISL_419180|2020-03-22	2020-03-22	France	
hCoV-19/France/ARA12265/2020|EPI_ISL_419186|2020-03-22	2020-03-22	France	
hCoV-19/Taiwan/TSGH-07/2020|EPI_ISL_427394|2020-03-22	2020-03-22	Taiwan	
hCoV-19/Belgium/ULG-9739/2020|EPI_ISL_418663|2020-03-22	2020-03-22	Belgium	
hCoV-19/DRC/248/2020|EPI_ISL_420035|2020-03-22	2020-03-22	DRC	
hCoV-19/France/IDF3230/2020|EPI_ISL_420063|2020-03-22	2020-03-22	France	
hCoV-19/Belgium/ULG-9725/2020|EPI_ISL_418659|2020-03-22	2020-03-22	Belgium	
hCoV-19/Australia/VIC285/2020|EPI_ISL_419976|2020-03-22	2020-03-22	Australia	
hCoV-19/Australia/VIC283/2020|EPI_ISL_419974|2020-03-22	2020-03-22	Australia	
hCoV-19/Australia/VIC286/2020|EPI_ISL_419977|2020-03-22	2020-03-22	Australia	
hCoV-19/Australia/SAP044/2020|EPI_ISL_451084|2020-03-22	2020-03-22	Australia	
hCoV-19/France/ARA12384/2020|EPI_ISL_420605|2020-03-22	2020-03-22	France	
hCoV-19/Australia/SAP127/2020|EPI_ISL_451136|2020-03-22	2020-03-22	Australia	

GISAID identifier of genomes that belong to 20A/15324T	collection date	Country	Canton if in Switzerland
hCoV-19/France/ARA12250/2020|EPI_ISL_419182|2020-03-22	2020-03-22	France	
hCoV-19/Luxembourg/LNS6277439/2020|EPI_ISL_429762|2020-03-22	2020-03-22	Luxembourg	
hCoV-19/Scotland/EDB112/2020|EPI_ISL_433407|2020-03-22	2020-03-22	Scotland	
hCoV-19/Scotland/EDB111/2020|EPI_ISL_425899|2020-03-22	2020-03-22	Scotland	
hCoV-19/DRC/253/2020|EPI_ISL_420838|2020-03-22	2020-03-22	DRC	
hCoV-19/Brazil/CV49/2020|EPI_ISL_429702|2020-03-22	2020-03-22	Brazil	
hCoV-19/Belgium/EBN-0323116/2020|EPI_ISL_420373|2020-03-23	2020-03-23	Belgium	
hCoV-19/Netherlands/ZuidHolland_55/2020|EPI_ISL_422923|2020-03-23	2020-03-23	Netherlands	
hCoV-19/Belgium/CJM-0323175/2020|EPI_ISL_420432|2020-03-23	2020-03-23	Belgium	
hCoV-19/England/SHEF-C83ED/2020|EPI_ISL_432864|2020-03-23	2020-03-23	England	
hCoV-19/Belgium/MC-0323129/2020|EPI_ISL_420386|2020-03-23	2020-03-23	Belgium	
hCoV-19/Belgium/NA-0323141/2020|EPI_ISL_420398|2020-03-23	2020-03-23	Belgium	
hCoV-19/Luxembourg/LNS0814393/2020|EPI_ISL_429766|2020-03-23	2020-03-23	Luxembourg	
hCoV-19/France/HF3677/2020|EPI_ISL_428359|2020-03-23	2020-03-23	France	
hCoV-19/France/ARA12485/2020|EPI_ISL_420607|2020-03-23	2020-03-23	France	
hCoV-19/France/ARA12524/2020|EPI_ISL_420609|2020-03-23	2020-03-23	France	
hCoV-19/France/ARA12915/2020|EPI_ISL_420618|2020-03-23	2020-03-23	France	
hCoV-19/France/ARA12973/2020|EPI_ISL_420619|2020-03-23	2020-03-23	France	
hCoV-19/France/IDF3235/2020|EPI_ISL_420064|2020-03-23	2020-03-23	France	
hCoV-19/Vietnam/HCMC-05006/2020|EPI_ISL_498177|2020-03-23	2020-03-23	Vietnam	
hCoV-19/Belgium/GM-0323139/2020|EPI_ISL_420396|2020-03-23	2020-03-23	Belgium	
hCoV-19/Belgium/DB-0323140/2020|EPI_ISL_420397|2020-03-23	2020-03-23	Belgium	
hCoV-19/Belgium/UGent-117/2020|EPI_ISL_475077|2020-03-23	2020-03-23	Belgium	
hCoV-19/Luxembourg/LNS8726389/2020|EPI_ISL_429776|2020-03-23	2020-03-23	Luxembourg	
hCoV-19/Switzerland/GE2759/2020|EPI_ISL_429214|2020-03-23	2020-03-23	Switzerland	Geneva
hCoV-19/Scotland/GCVR-16FFD4/2020|EPI_ISL_459547|2020-03-23	2020-03-23	Scotland	
hCoV-19/Luxembourg/LNS0709551/2020|EPI_ISL_429781|2020-03-23	2020-03-23	Luxembourg	
hCoV-19/Belgium/LPDC-0323115/2020|EPI_ISL_420372|2020-03-23	2020-03-23	Belgium	
hCoV-19/Australia/SAP048/2020|EPI_ISL_451087|2020-03-23	2020-03-23	Australia	
hCoV-19/Australia/VIC312/2020|EPI_ISL_420003|2020-03-23	2020-03-23	Australia	
hCoV-19/Australia/VIC302/2020|EPI_ISL_419993|2020-03-23	2020-03-23	Australia	
hCoV-19/France/IDF3324/2020|EPI_ISL_421512|2020-03-23	2020-03-23	France	
hCoV-19/France/IDF3359/2020|EPI_ISL_443301|2020-03-23	2020-03-23	France	
hCoV-19/France/IDF3518/2020|EPI_ISL_428352|2020-03-23	2020-03-23	France	
hCoV-19/France/ARA12996/2020|EPI_ISL_420620|2020-03-23	2020-03-23	France	
hCoV-19/Luxembourg/LNS7299024/2020|EPI_ISL_429775|2020-03-23	2020-03-23	Luxembourg	
