# Supplementary material for: SARS-CoV-2 outbreak in a tri-national urban area is dominated by a B.1 lineage variant linked to a mass gathering event
Source: PLoS Pathog. 2021 Mar 19;17(3):e1009374. doi: 10.1371/journal.ppat.1009374 (PMC8011817; doi:10.1371/journal.ppat.1009374)
Supplement: S6 Table — Inferred by contributors to https://github.com/W-L/ProblematicSites_SARS-CoV2. (PDF) [file ppat.1009374.s012.pdf]

Table S6. Nucleotide position in relation to the Wuhan-Hu1 reference sequence that were masked for phylogenetic inferences, due to homoplasies. Inferred by contributors to https://github.com/W-L/ProblematicSites_SARS-CoV2**.**

Start position	End position
635	635
2091	2091
2094	2094
3145	3145
3564	3564
4050	4050
5736	5736
6869	6869
8022	8022
8790	8790
10129	10129
11074	11074
11083	11083
11535	11535
13402	13402
13408	13408
13476	13476
13571	13571
14277	14277
15922	15922
16887	16887
19484	19484
21575	21575
22335	22335
24389	24389
24390	24390
24933	24933
26549	26549
29037	29037
29553	29553
