# Supplementary material for: SARS-CoV-2 outbreak in a tri-national urban area is dominated by a B.1 lineage variant linked to a mass gathering event
Source: PLoS Pathog. 2021 Mar 19;17(3):e1009374. doi: 10.1371/journal.ppat.1009374 (PMC8011817; doi:10.1371/journal.ppat.1009374)
Supplement: S1 Text — (PDF) [file ppat.1009374.s014.pdf]

## Supplementary Results

### Basel samples in phylogenetic global context continued

*Cluster B.1.5*

Isolates that are assigned to lineage B.1.5 make up 2.6% of USB isolates (**Figure S6B**). They all share the A20268G mutation. Three unresolved branches defined by at least one additional mutation each, diverge from the internal node consisting of, from top to bottom, six (C25658T), six (C28854T), and one (G25483A, C4893T, C23380A, C26509T [mutations in order of temporal appearance]) Basel area isolates. Individual isolates can exhibit one to three additional mutations. Isolates date from March 13$^{th}$ to March 23$^{rd}$ with an inferred node age of February 19$^{th}$ (CI: January 13$^{th}$-Ferbuary 20$^{th}$, 2020). No social connections for transmission patterns within each branch could be inferred from the available patient data. Searching the clade defining mutations in the nextstrain.org phylogeny we gain the following insights. Mutation C25658T (plus the clade defining A20268G) is found in one isolate (Oman/RESP-20-6701/2020 from March 28$^{th}$); C28854T is found 17 isolates, two of which show no additional mutations (Norway/2088/2020 from March 17$^{th}$, Latvia/045/2020 from March 22$^{nd}$) just like two of our isolates (42193056, 42189239). Derived isolates originate from Switzerland, Scotland, Romania, USA, Taiwan, and England. Mutation G25483A recorded in a single isolate (42202280) is not currently reported in the nextstrain.org phylogeny.

*Cluster B.1.8*

Isolates that are assigned to lineage B.1.8 make up 0.7% of USB isolates (**Figure S6C**). They all share the A24862G mutation. Isolates date from March 14$^{th}$ to March 22$^{nd}$ with an inferred internal node age of February 1$^{st}$ (CI: January 12$^{th}$-March 8$^{th}$, 2020). Two isolates (42191012, 42202147) exhibit the identical mutational pattern (additional T658C, C28829T) but have no known epidemiological link. Our own global comparison identified an isolate from Germany (Germany/NRW-34/2020 from March 16$^{th}$) that exhibits the same mutations. Searching the clade defining mutation in the nextstrain.org phylogeny does not yield better insights into the evolution of the lineages as no isolates with the same pattern could be identified.

*Family clusters within lineage B.2*

We identified eight genomes that were assigned to lineage B.2 (**Figure S6D**). They all share the G26144T mutation that translates into amino acid change ORF3a-G251V and date from March 13$^{th}$ to March 22$^{nd}$ with an inferred internal node age of January 15$^{th}$ (CI: January 13$^{th}$-January 18$^{th}$, 2020). This cluster harbours two household transmission clusters: *Family 2* with two members and *Family 3* with three members. These two clusters share C14805T (synonymous in *ORF1ab*) and exhibit unique

additional mutations C9319T (synonymous in *ORF1ab*) and G12278T (ORF1ab-A4005S), G26730T (M-V70F), G29414T (N-A381S), respectively. We find no evidence of further community transmission. These mutational combinations are not currently represented in the full global phylogeny (nextstrain.org), suggesting that quarantine measures were effective in these cases and inhibited further transmission events.
